# M6A-modified circRBM33 promotes prostate cancer progression via PDHA1-mediated mitochondrial respiration regulation and presents a potential target for ARSI therapy

**DOI:** 10.7150/ijbs.77133

**Published:** 2023-03-05

**Authors:** Chuanfan Zhong, Zining Long, Taowei Yang, Shuo Wang, Weibo Zhong, Feng Hu, Jeremy Yuen-Chun TEOH, Jianming Lu, Xiangming Mao

**Affiliations:** 1 Department of Urology, Zhujiang Hospital, Southern Medical University, Guangzhou, China.; 2 Department of Urology, Kiang Wu Hospital, 85-87 R.de Coelho do Amaral, Macao, China.; 3 S. H. Ho Urology Centre, Department of Surgery, The Chinese University of Hong Kong, Hong Kong, China.; 4 Department of Andrology, Guangzhou First People's Hospital, Guangzhou Medical University, Guangzhou, China.

**Keywords:** Prostate cancer, N6-methyladenosine, circRBM33, mitochondrial respiration, ARSI therapy

## Abstract

N6-Methyladenosine (m6A) is the most prevalent RNA modification in various types of RNA, including circular RNAs (circRNAs). Mounting evidence has shown that circRNAs may play critical roles in diverse malignancies. However, the biological relevance of m6A modification of circRNAs in prostate cancer (PCa) remains unclear and needs to be elucidated. Our data showed that circRBM33 was m6A-modified and was more highly expressed in PCa cells than in normal cells/tissues. The *in vitro* and* in vivo* experiments showed that downregulation/upregulation of circRBM33 inhibited/promoted tumour growth and invasion, respectively. Decreasing m6A levels rescued the tumour-promoting effect of circRBM33. Additionally, once modified by m6A, circRBM33 interacts with FMR1 by forming a binary complex that sustains the mRNA stability of PDHA1, a downstream target gene. Suppressed/overexpressed circRBM33 lowered/enhanced the ATP production, the acetyl-CoA levels and the NADH/NAD^+^ ratio. Moreover, depletion of circRBM33 significantly increased the response sensitivity to androgen receptor signalling inhibitor (ARSI) therapy, including enzalutamide and darolutamide, in prostate tumours. Our study suggested that the m6A-mediated circRBM33-FMR1 complex can activate mitochondrial metabolism by stabilizing PDHA1 mRNA, which promotes PCa progression, and can attenuate circRBM33 increased ARSI effectiveness in PCa treatment. This newly discovered circRNA may serve as a potential therapeutic target for PCa.

## Introduction

Prostate cancer (PCa) ranks first in incidence and second in mortality among male malignancies in the USA 1. Androgen deprivation therapy (ADT), in which androgen levels are lowered by surgery or drugs, is the mainstay of treatment for aggressive PCa 2, 3. However, almost all prostate cancers will inevitably develop into castration-resistant prostate cancer (CRPC) approximately 18 months after ADT due to abnormal reactivation of the androgen receptor signalling pathways 4. Fortunately, androgen receptor signalling inhibitors (ARSIs) involving androgen synthesis inhibitors (Abiraterone et al.) and AR antagonists (enzalutamide, darolutamide, apalutamide, etc.) have been applied to CRPC treatment, which significantly extends these patients' overall survival 5, 6. Even so, CRPC will ultimately become insensitive to ARSI therapy because of AR splice variants or bypass activation 7. Thus, new therapeutic strategies for CRPC are urgently needed.

N6-methyladenosine (m6A) is the most common mRNA modification in mammals 8. It is a reversible epigenetic regulatory mechanism that contains three groups of proteins: writers (methyltransferases), erasers (demethylases) and readers (m6A recognition proteins). METTL3 is the core catalytic unit of the methyltransferase complex, which was reported to regulate proliferation, metastasis, angiogenesis and metabolism in a variety of cancers, including PCa 9-11. FTO, the first confirmed demethylase of m6A, was initially shown to play an important role in the aggressiveness of leukaemia and was sequentially proven to exert different roles in a variety of solid cancers 12-14. Additionally, “readers” also perform a wide range of functional effects on their corresponding binding RNA, such as stability, degradation, and translation 15. For example, YTHDF1 can bind to the m6A-modified site of EIF3C mRNA and augment its translation, thereby promoting tumorigenesis and metastasis of ovarian cancer 16. In addition to modifying mRNA, m6A was also found in many noncoding RNA types, such as circular RNA (circRNA) 17. CircRNA is a kind of back-spliced noncoding RNA that was previously viewed as an erroneously processed product 18. To date, accumulating studies have revealed that circRNAs participate in the development and pathogenesis of various diseases through different means, such as acting as miRNA sponges, interacting with proteins or even translating novel peptides 19, 20. It has been reported that m6A modification not only helps synthesize circRNAs and nucleocytoplasmic transport but also facilitates circRNA-protein interactions and even endows circRNAs with translational competence 21. Conversely, circRNA can regulate m6A modification as well, e.g., circ0008399 interacts with WTAP to promote the assembly of the m6A complex and enhance cisplatin resistance in bladder cancer 22. Therefore, the interplay between m6A modification and circRNA is intricate and requires further exploration and research.

Historically, mitochondrial metabolism was thought to play an inconsequential role in the rapid proliferation of cancers 23. This may stem from a classical theory raised by Otto Warburg in the 1920s that cancer cells were inclined to take up glucose to produce lactate in the presence of oxygen due to the enormous requirement for adenosine triphosphate (ATP). This metabolic process is called aerobic glycolysis 24. However, emerging evidence has confirmed that mitochondrial respiration is also crucial for tumorigenesis and metastasis 25. The tricarboxylic acid (TCA) cycle is a “metabolic hub” providing the major intermediates that are critical for various metabolic processes, including lipogenesis, amino acid metabolism, and nucleotide biosynthesis 26. Thus, in addition to glycolysis, cancer cells also engage mitochondrial oxidative phosphorylation (ox-pho) to support tumour growth. It has been reported that tumour cells enhance the ox-pho process while switching from a proliferative to an invasive state 27, 28. A recent study revealed that ox-pho was substantially increased in CPRC cells compared to hormone-sensitive prostate cancer (HSPC) cells 29.

In our study, through comprehensive analysis of public (GSE113124) and our own m6A-RIP sequencing data, we screened hsa_circ_0001771 (circRBM33) as our target gene, and it is a m6A-modified circRNA. After verification of the basic characteristics of circRBM33, we conducted *in vitro* and *in vivo* experiments, and the results showed that circRBM33 plays a pro-tumor role in PCa and that reducing m6A levels attenuated the tumour-promoting effect of circRBM33. Additionally, we applied clinicopathological analysis of circRBM33. It was demonstrated that circRBM33 positively correlated with Gleason score (GS), and high expression of circRBM33 predicts poor biochemical recurrence (BCR)-free survival in patients with PCa. Mechanistically, circRBM33 interacted with FMR1 to form a binary complex in a m6A manner and then bound PDHA1 mRNA to enhance its stability and increase translational output, thereby strengthening ox-pho and promoting PCa growth and metastasis. Furthermore, we examined whether circRBM33 is related to the ARSI therapy response, and the results revealed that knockdown of circRBM33 increased PCa sensitivity to enzalutamide and darolutamide *in vitro* and *in vivo.*

## Methods and materials

### Ethics Approval

The current study was approved by the Ethics Committee of Zhujiang Hospital, Southern Medical University. All animal experiments were approved by the Animal Care Committee of Zhujiang Hospital of Southern Medical University.

### Cell Culture

The human prostatic epithelial cell line RWPE-1 was obtained from iCell Bioscience. Human prostate cancer cell lines, including LNCaP, C4-2, PC-3, DU145 and 22Rv1, were purchased from BeNa Culture Collection. RWPE-1 cells were cultured in keratinocyte serum-free medium (K-SFM) supplemented with 0.05 mg/ml bovine pituitary extract and 5 ng/ml human recombinant epidermal growth factor. LNCaP, C4-2, and 22Rv1 cells were cultured in RPMI-1640 medium with 10% foetal bovine serum (FBS) and 1% penicillin‒streptomycin solution. PC-3 and DU145 cells were cultivated in DMEM with 10% FBS and 1% penicillin‒streptomycin solution. All cell lines were cultured at 37 °C with 5% CO_2_.

### Plasmids, Oligo RNA and Lentivirus

Full-length circRBM33 was inserted into the pLC5-ciR plasmid (Geneseed) for intracellular overexpression. Lentivirus carrying encoding short hairpin RNAs (shRNA) targeting circRBM33 was synthesized by GeneChem. pcDNA3.1 carrying FMR1 and pLKO.1 plasmids carrying shRNA targeting METTL3 were synthesized by Tsingke Biotechnology. Lentivirus assembly was performed in 293T cells using the enveloped plasmid VSV-G and backbone plasmid PAX2 with polyethylenimine (PEI). Small interfering RNA (siRNA) targeting FMR1 and fluorescent and biotinylated probes targeting circRBM33 were synthesized by Genepharm. Primers were synthesized by Tianyi Huayuan. The sequence information of all primers and probes is listed in Supplementary Table 1.

### Quantitative Real-time Polymerase Chain Reaction (qRT‒PCR)

Total RNA was isolated using TRIzol reagent (Invitrogen) and quantified by NanoDrop. RNA reverse transcription was carried out using TransScript® Uni One-Step gDNA Removal and cDNA Synthesis SuperMix (Transgen), and qPCR assays were performed in StepOnePlus Real Time PCR system (Applied Biosystems) using PerfectStart® Green qPCR SuperMix (Transgen).

### RNA Stability Assays

For evaluation of RNA stability between circRBM33 and linearBM33, PCa cells were treated with 2 mg/ml actinomycin D (Sigma, USA) at a gradient time point including 0, 6, 12 and 24 hours, and then total RNA was collected from the corresponding cell samples or total RNA from untreated cells was digested by RNase R (2 U/μg RNA) for 30 min. For evaluation of RNA stability variation of PDHA1, transfected cells were treated with 2 mg/ml actinomycin D at 0, 3, 6 and 9 hours to obtain corresponding total RNA. Finally, the expression levels of the above genes were measured by qPCR.

### Nucleocytoplasmic RNA Separation

The nuclear and cytoplasmic fractions of PCa cells were separated using the PARIS™ Kit (Ambion, Lifetechnologies) according to the manufacturer's instructions.

### Antibodies and Western blot

Anti-FMR1 antibody (#4317), anti-HK2 antibody (#2867), anti-PFKP antibody (#12746), anti-PKM1/2 antibody (#3186), and anti-LDHA antibody (#3582) were purchased from Cell Signaling Technology. Anti-TKT antibody (11039-1-AP), anti-G6PD antibody (25413-1-AP), anti-β-actin antibody (66009-1-Ig) and Anti-AR antibody (22089-1-AP) were purchased from Proteintech. Anti-METTL3 (A8370) was purchased from ABclonal Technology. Anti-RBM33 antibody (bs-21295R) was purchased from Bioss. Anti-N6-methyladenosine antibody (ab208577) was purchased from Abcam. Anti-AR-v7 antibody (T55510) was purchased from Abmart. Cell lysates were obtained using RIPA buffer, isolated by 10% sodium dodecyl sulphate‒polyacrylamide gel electrophoresis and transferred onto a 0.45 μM polyvinylidene fluoride membrane. After immersion in 5% nonfat milk for 1 h, the membranes were incubated with primary antibodies at 4 °C overnight, followed by incubation with secondary antibodies for 1 h at room temperature. A chemiluminescence imaging system (CLiNX ChemiScope Touch, Shanghai) was used to observe the protein expression level.

### Cell Viability, Colony Formation and Invasion assays

For cell viability assays, 2000 transfected cells with or without STM2457 (S9870, Selleck) treatment were seeded into a 98-well plate and examined by a VICTOR Nivo^TM^ Multimode Plate reader (PerkinElmer) using a CCK-8 kit (Meilune). For the clone forming assay, 1000 transfected cells were seeded into 6-well or 12-well plates, cultured in an incubator for 14 days and stained with 1% crystal violet solution. For invasion assays, 6×10^4^ transfected cells were seeded into the upper chamber of the transwell after Matrigel matrix (354234, Corning) layering, cultivated in an incubator for 24 or 48 h and stained with 1% crystal violet solution. For ARSI sensitivity assays, transfected cells were treated with enzalutamide (S1250, Selleck) or darolutamide (HY-16985, MCE) at a concentration gradient of 10-100 μM for 48 h and then evaluated by CCK-8. Each experiment was independently repeated in triplicate.

### Fluorescence in Situ Hybridization (FISH) and Immunofluorescence (IF)

Cy3-marked probes of circRBM33 and 18S were synthesized by GenePharm Company. For the FISH assay, we performed this experiment using the RiboTM Fluorescent In Situ Hybridization Kit (RIBOBIO, C10910). Briefly, PCa cells were fixed with 4% paraformaldehyde followed by perforation with precooled 1% Triton. Then, the cells were incubated with prehybridization buffer at room temperature for 30 min and incubated with hybridization buffer mixed with 20 μM probes at 37 °C overnight. Ultimately, the cells were washed using washing buffer with 4 X SSC, 0.1% Tween-20, 2 X SSC and 1 X SSC sequentially and stained with anti-fluorescence quenching solution (containing DAPI) (P0131). For colocalization observation of circRBM33 and FMR1, FISH was conducted as described above without DAPI staining. Cells were blocked with 1% bovine serum albumin (BSA) solution at room temperature for 30 min prior to incubation with the primary anti-FMR1 antibody at 4 °C overnight. Subsequently, the cells were incubated with CoraLite488-conjugated goat anti-rabbit IgG (H+L) secondary antibody (Proteintech, SA00013), followed by staining with DAPI. Finally, colocalization was observed using a confocal imaging system (ZEISS, LSM 900 with Airyscan 2).

### Immunohistochemistry (IHC)

Tissue microarrays (TMA; HProA060PG01) were purchased from Shanghai Outdo Biotech Limited Company. The xenografts were fixed in 4% paraformaldehyde prior to embedding in paraffin. Each tissue block was cut into 4-μm-thick slices. TMA or xenograft sections were first deparaffinized using xylene and then rehydrated by immersion in 100% twice, 95% twice, 90%, 80%, and 70% ethanol solutions. For histological observation, slices were examined using a Haematoxylin-Eosin/HE Staining Kit (Solarbio, G1120) according to the manufacturer's instructions. For immunohistochemistry, antigens in slices or TMAs were retrieved using citrate antigen retrieval solution. Subsequently, the internal peroxidase activity was blocked with 1% H_2_O_2_ solution, and then the slices were blocked with nonimmune goat serum. Slices were incubated with the primary antibody at 4 °C overnight and incubated with biotin secondary antibody at room temperature for 30 min, followed by incubation with streptavidin-conjugated HRP at room temperature for 15 min. After that, HRP activity was examined by diaminobenzidine tetrahydrochloride (DAB), and nuclei were detected by haematoxylin staining. Finally, the slices were dehydrated in 70%, 80%, 90%, 95%, and 100% ethanol solutions, cleared with xylene and sealed using neutral gum. Slices were scanned by a pathological section scanner (Leica, SDPTOP HS6). The antibodies used were as follows: anti-Ki67 antibody (Abcam, ab15580), anti-FMR1 antibody (Proteintech, 13755-1-AP), and anti-PDHA1 antibody (Proteintech, 18068-1-AP).

### Xenograft Experiment

Four-week-old male nude BALB/c mice were purchased from SPF (Beijing) Biotechnology Co., Ltd. A total of 5×10^6^ cells expressing vector or circRBM33 (or sh-circRBM33) were subcutaneously injected into both back sides of nude mice. For drug treatment, mice bearing transplanted tumours were randomly divided into two groups when the tumour volume reached 100-150 mm^3^ and then treated with vehicle or 20 mg/kg enzalutamide (or 50 mg/kg darolutamide) orally for 28 days. Tumour volume was measured every 3 days by calculating 0.5×length×width^2^, and the tumour weight was finally measured after sacrifice.

### Acetyl-CoA and NAD^+^/NADH Ratios and ATP examination

NAD^+^/NADH ratios (Beyotime, S0175) were measured by the NAD^+^/NADH Assay Kit with WST-8, ATP production levels (Beyotime, S0027) were measured by the Enhanced ATP Assay Kit, and acetyl-CoA levels (MEIMIAN, AB-3528A) were measured by the Human Acetyl-CoA (A-CoA) ELISA Kit. These assays were carried out according to the manufacturer's instructions. Each assay was independently repeated in triplicate.

### Seahorse Metabolic Flux Assay

The oxygen consumption rate (OCR) was detected by an XF-24 Extracellular Flux Analyser (Seahorse Bioscience). Briefly, 2x10^4^ transfected PCa cells were seeded into the indicated well in XF24-well cell culture microplates (Seahorse Bioscience) for 24 h. Then, the adherent cells were washed with base medium and incubated in a CO^2^-free incubator at 37 °C for at least 1 h. After calibration of temperature and pH equilibration, the microplates were loaded into the analyser for assessment. Mitochondrial respiration was measured using the Seahorse XF Cell Mito Stress test kit (Agilent) following the manufacturer's instructions. Compound injections of oligomycin, FCCP and rotenone/antimycin A were applied on the microplate.

### Methylated RNA Immunoprecipitation (meRIP) and RIP Assay

MeRIP and RIP assays were conducted using a Methylated RNA Immunoprecipitation Kit (BersinBio, Bes5203) and RNA Immunoprecipitation Kit (BersinBio, Bes5101). For the meRIP assay, total RNA was isolated by TRIzol and fragmented to approximately 300 bp by an ultrasonic cell disruptor. Then, the fragmented RNA was coimmunoprecipitated with anti-N6-methyladenosine antibody in the vertical rotator at 4 °C for 4 h, incubated with protein A/G beads for 1 h, and eluted by proteinase K at 55 °C for 45 min to acquire the m6A-modified RNA. For the RIP assay, the collected cell pellets were lysed in lysis buffer on ice, and DNA was removed with DNase. Subsequently, the cell lysate was immunoprecipitated with anti-AGO2 or anti-FMR1 antibody at 4 °C for 4 h, incubated with protein A/G beads for 1 h and eluted using proteinase K at 55 °C for 45 min. RNA samples were finally examined by qPCR.

### Chromatin Isolation by RNA Purification (ChIRP) Assay

The ChIRP assay was performed using a Chromatin Isolation by RNA Purification (ChIRP) Kit (BersinBio, Bes5104) according to the manufacturer's instructions. Briefly, the collected cell pellet was cross-linked by 4% formaldehyde solution and neutralized by glycine at room temperature. Then, the cell pellet was lysed by swelling buffer and nuclear lysis buffer with protease and RNase inhibitors. Subsequently, the cell lysate was fragmented by sonication and precleared with agarose beads at 4 °C. The biotinylated probes targeting the back-splice junction of circRBM33 and the negative control probes were hybridized to cell lysates prior to incubation with streptavidin beads. Finally, RNA and protein samples were eluted and validated by qPCR and WB, respectively.

### Public Data Acquisition and Analysis

The circRNA-seq data of circRNA in 144 patients with PCa were acquired from the GEO database (accession: GSE113124) and are listed in Supplementary Table 2. The Kaplan‒Meier (KM) plot of DFS analysis in PCa patients based on FMR1 expression level was processed and downloaded from the Gepia 2 website (http://gepia2.cancer-pku.cn/#index). The encoding potential of circRBM33 was predicted by CircRNADb (http://reprod.njmu.edu.cn/cgi-bin/circrnadb/circRNADb.php). The predicted proteins interacting with circRBM33 were acquired from catRAPID (http://service.tartaglialab.com/page/catrapid_group) and CircInteractome (https://circinteractome.nia.nih.gov/). Graphical abstract was created with BioRender (https://biorender.com).

### Statistical Analysis

All statistical data were processed and analysed by GraphPad Prism 8.0 Software. Analytical results are presented as the mean ± standard error. Student's t test was used to test the significance of differences in two grouped data, and one-way analysis of variance (ANOVA) was applied to three or more independent groups. The chi-square test was utilized for the clinicopathologic analysis. Statistically significant p values are represented as *p <0.05, **p < 0.01 and ***p < 0.001. ****p < 0.0001.

## Results

### CircRBM33 is m6A-modified and Predicts a Poor Prognosis in PCa

**Figure 1:**
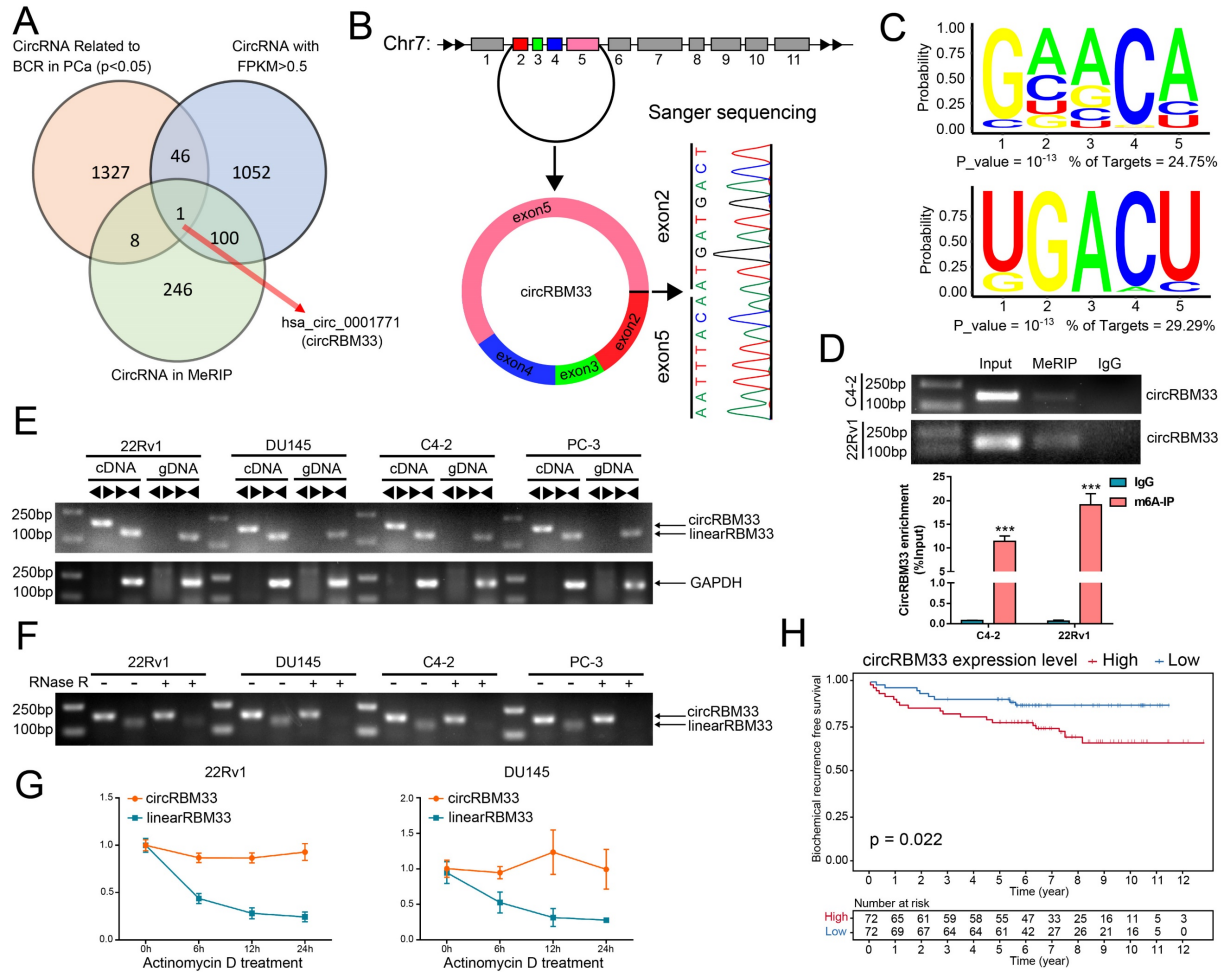
CircRBM33 is m6A-modified and predicts a poor prognosis in PCa. (A) The Venn diagram shows the only overlapping circRNA among the BCR-related circRNAs in PCa, circRNAs with FPKM > 0.5, and circRNAs in the MeRIP analysis. (B) Sanger sequencing confirms the back-splicing site of circRBM33. (C) The motif analysis predicts the potential m6A-modified sites in circRBM33. (D) The MeRIP assay examines the is m6A-modified status of circRBM33 (E) Divergent and convergent primers amplification assays confirms the derivation of circRBM33. (F) The RNase R assay confirms the stability of circRBM33 and linearRBM33. (G) The Actinomycin D assay detects the stability disparity between circRBM33 and linearRBM33. (H) The Kaplan-Meier survival curves display the prognosis value of circRBM33 in PCa.

We integrated three data profiles to determine both m6A-related and prognosis-associated circRNAs in PCa (Figure 1A). First, we obtained the expression matrix of all circRNAs in PCa (GSE113124) and screened out those circRNAs with low expression (FPKM < 0.5). Then, we investigated the follow-up data (GSE113124) to select the circRNAs related to BCR in PCa, and 1382 circRNAs were statistically significant (p < 0.05). With discretion, we performed MeRIP sequencing using PCa cell samples to investigate the m6A-related circRNA profile, and 355 circRNAs tended to be m6A-related (Table S2). Next, we drew a Venn diagram to focus on the overlap, and consequently, a m6A-related circRNA with prognostic value, circRBM33, known as hsa_circ_0001771, showed up in the overlap zone. As shown in Figure 1B, the gene that encodes circRBM33 falls at chromosome 7, and exons 2, 3, 4, and 5 join together to generate circRBM33 with the help of back-splicing between exon 2 and exon 5. Thus, we then performed Sanger sequencing to confirm the putative head-to-tail conjunction between exon 2 and exon 5. Subsequently, we conducted a motif analysis from the MeRIP data to locate the putative m6A-modified sites, and two motifs were likely to be potential targets (Figure 1C). Furthermore, we preliminarily inspected whether circRBM33 is m6A-modified with a meRIP assay. As shown in Figure 1D, circRBM33 appeared in the m6A-antibody channel and the input channel instead of the IgG channel in both PCa cell lines, indicating that circRBM33 is m6A-modified. To further ascertain the existence of circRBM33, we amplified the transcripts of RBM33 from cDNA (complementary DNA) and gDNA (genomic DNA) using two different kinds of primers, divergent and convergent, in four PCa cell lines (Figure 1E). Consistently, circRBM33 could only be amplified in cDNA instead of gDNA with the help of divergent primers in each PCa cell line. On the one hand, we performed a ribonuclease R (RNase R) assay for a stability comparison between circRBM33 and linearRBM33 (Figure 1F). Regarding the results of gel electrophoresis, circRBM33 was more stable than linear RBM33 after RNase R treatment. On the other hand, we also implemented an actinomycin D assay to display the better stability of circRBM33, and the results were consistent with the above results (Figure 1G). Following validation of the authenticity of circRBM33, we investigated whether it is related to favourable or poor prognosis in PCa. Then, we performed KM survival analysis to measure its predictive power using a circRNA database (Figure 1H). The PCa patients were divided into two subgroups by the median circRBM33 expression: the high circRBM33-expression group and the low circRBM33-expression group. Consequently, patients with a higher expression level of circRBM33 had worse BCR-free survival than those with a low expression level of circRBM33. We also examined the relationship between circRBM33 expression and some clinicopathological characteristics in PCa. Supplemental Table 3 shows that circRBM33 expression levels increased slightly as the GS increased and the tumour progressed, although no statistical significance was found. In short, circRBM33 presents itself as m6A-modified and serves as a BCR prognosis indicator in PCa.

### m6A-modified CircRBM33 Promotes PCa Proliferation and Invasion *in vitro*

**Figure 2:**
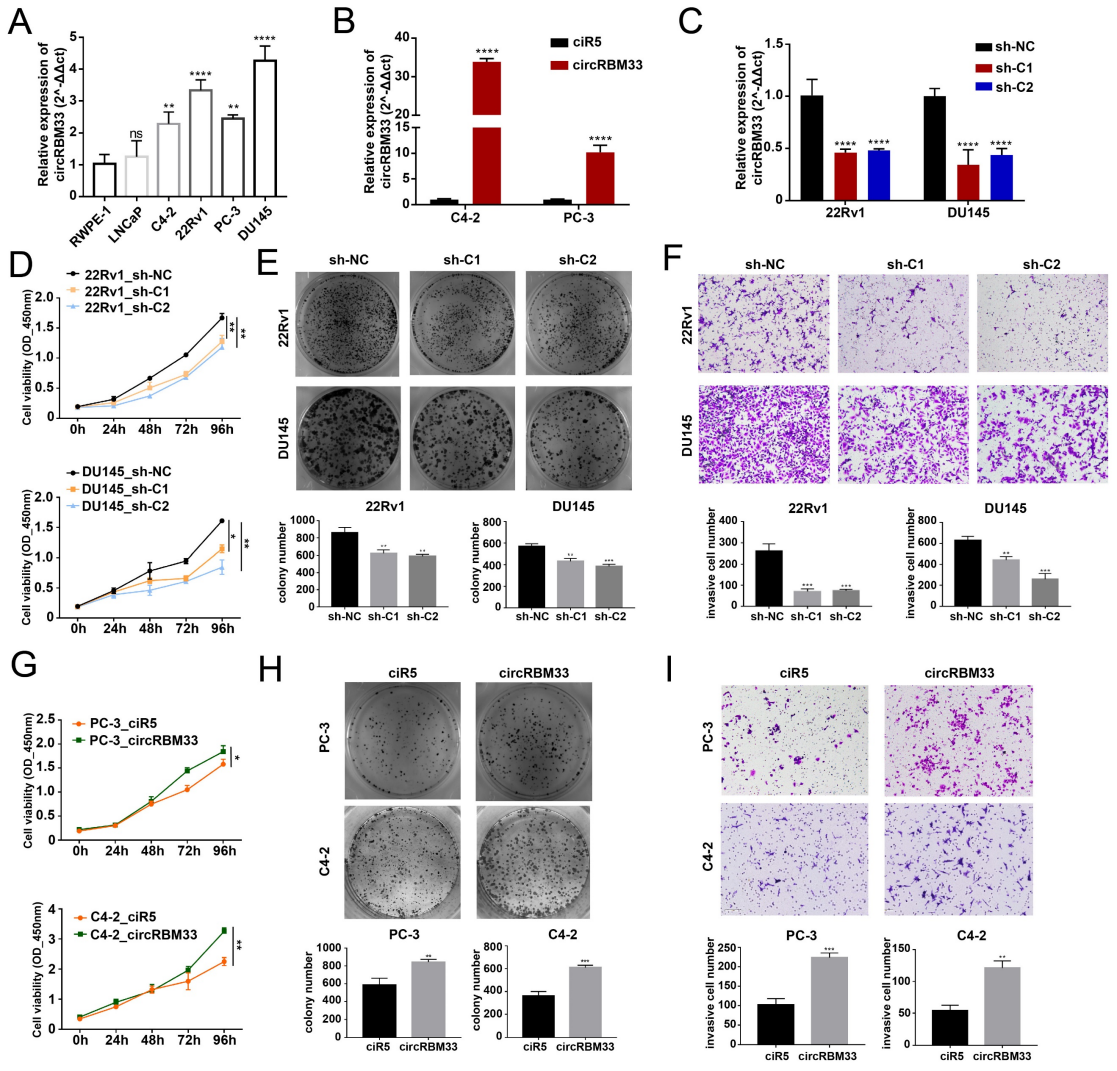
CircRBM33 promotes PCa's proliferation and invasion *in vitro.* (A) qRT-PCR confirms circRBM33 expression in five PCa cell lines (LNCaP, C4-2, 22Rv1, PC-3, and DU145), as well as RWPE-1. (B) qRT-PCR confirms the transfection efficiency of overexpressing circRBM33 in C4-2 and PC-3 cell lines. (C) qRT-PCR confirms the transfection efficiency of silencing circRBM33 (shC1 and shC2) in 22Rv1 and DU145 cell lines. (D) The CCK-8 assay measures the cell viability in circRBM33-silenced and negative control PCa cells. (E) The plate colony formation assay detects the colony formation ability in circRBM33-silenced and negative control PCa cells. (F) The Transwell assay determines the invasiveness discrepancy between circRBM33-silenced and negative control PCa cells. (G) The CCK-8 assay measures the cell viability in circRBM33-upregulated and negative control PCa cells. (H) The plate colony formation assay detects the colony formation ability in circRBM33-overexpressed and negative control PCa cells. (I) The Transwell assay determines the invasiveness discrepancy between circRBM33-overexpressed and negative control PCa cells.

According to the KM analysis results above, circRBM33 tends to be a pro-cancer factor in PCa. Therefore, we conducted further experiments to confirm our findings. First, we confirmed circRBM33 expression in various PCa cell lines, including LNCaP, C4-2, 22Rv1, PC-3, and DU145, as well as an immortalized human prostate epithelial cell line (RWPE-1) (Figure 2A). As a result, circRBM33 is expressed at high levels in 22Rv1 and DU145 cells, with low expression in C4-2 and PC-3 cells. Accordingly, we chose to silence circRBM33 in 22Rv1 and DU145 cells, and in parallel, we overexpressed circRBM33 in C4-2 and PC-3 cells. We upregulated circRBM33 in both C4-2 and PC-3 cells via lentivirus vectors, with ciR5 acting as a negative control (Figure 2B). Likewise, we downregulated circRBM33 in 22Rv1 and DU145 cells (shC1 and shC2), with sh-NC serving as a negative control (Figure 2C). At the transcriptional level (Figure S1A), lentivirus transduction had solely an upregulating/downregulating effect on circRBM33 rather than linearRBM33. We also detected that RBM33 expression at the translational level was slightly changed in these well-constructed cell lines (Figure S1B). The CCK-8 cell viability assay showed that knocking down circRBM33 inhibited PCa cell proliferation (Figure 2D), and the plate colony formation assay demonstrated a similar result: downregulating circRBM33 attenuated PCa cell viability (Figure 2E). Apart from proliferation, the transwell assay indicated that silencing circRBM33 hindered PCa cell invasiveness (Figure 2F). In contrast, overexpressing circRBM33 stimulated PCa cell growth, as proven by the CCK-8 assay and plate colony formation assay (Figure 2G-H). Instead, upregulating circRBM33 increased PCa cell invasiveness (Figure 2I). Taken together, circRBM33 stimulated PCa proliferation and invasion *in vitro*. As mentioned above, circRBM33 was m6A-modified in PCa, and we subsequently tested the regulatory role of m6A in PCa progression. A METTL3-specific inhibitor (STM2457) was added to PC-3 and C4-2 cell lines, and dot blot assays were applied to examine the m6A level change. As depicted in Supplementary Figure 2A, 5 μM STM2457 thoroughly decreased the m6A level in these cell lines. Interestingly, lowered m6A levels significantly weakened circRBM33-induced proliferation and metastasis capability (Figure S2B-D).

### CircRBM33 Interacts with FMR1 in a m6A-Mediated Manner

**Figure 3:**
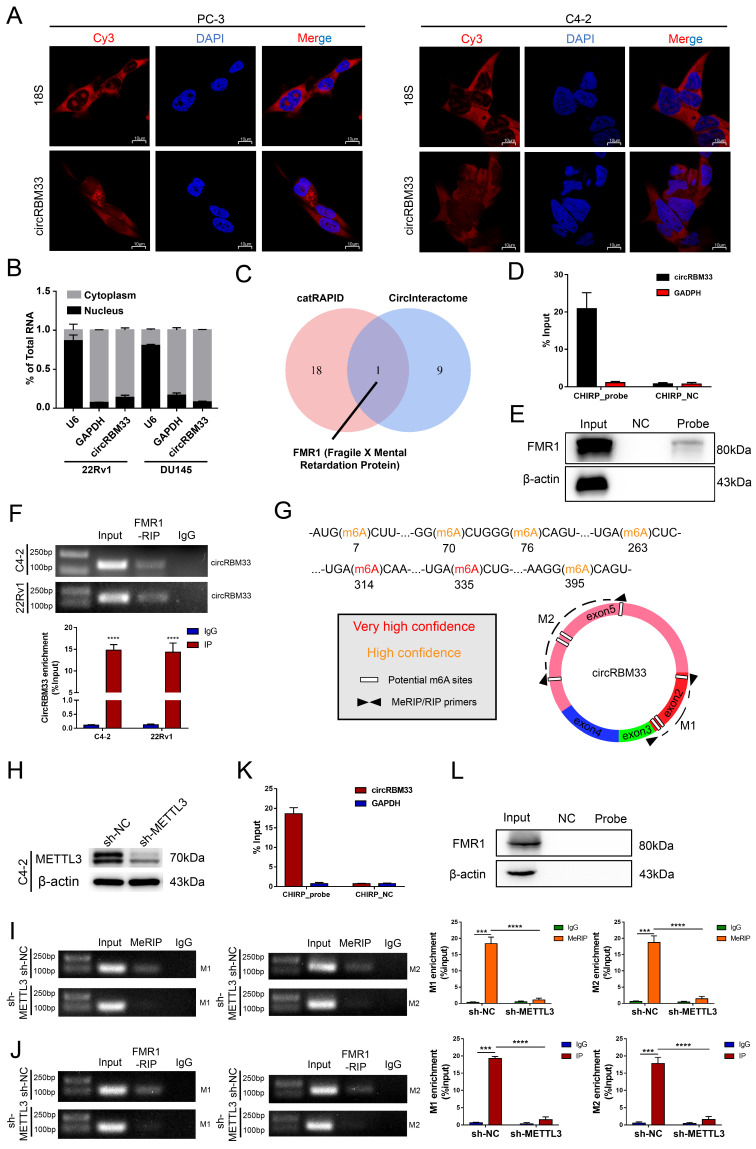
CircRBM33 interacts with FMR1 in m6A-mediated manner. (A) FISH assays show the subcellular localization of circRBM33 in PCa cells (PC-3 and C4-2) with 18S acting as a positive control. (B) The nuclear and cytoplasmic extraction experiments show the nucleus/cytoplasm proportion of circRBM33. (C) The protein that overlapped between the catRAPID database and the CircInteractome database. (D) qRT-PCR confirms the enrichment of the ChIRP probe designed to circRBM33. (E) WB confirms the pulldown of FMR1 by the ChIRP probe. (F) The RIP experiments show the enrichment of circRBM33 by FMR1. (G) A schematic representation of the potential m6A-modified sites in circRBM33. (H) Wb confirms the transfection efficiency of shRNA to METTL3. (I) MeRIP assay confirms the enrichment of circRBM33 by m6A in METTL3-knockdown and control groups. (J) FMR1-RIP assay detects the enrichment of circRBM33 by FMR1 in METTL3-knockdown and control groups. (K) D) qRT-PCR confirms the enrichment of the ChIRP probe designed to circRBM33 in METTL3-knockdown groups. (L) WB confirms the pulldown of FMR1 by the ChIRP probe under the downregulation of METTL3.

We then inspected how circRBM33 exerts its effects on PCa. The localization of circRBM33 indicates its regulatory mechanisms to some degree, so we first performed FISH assays to inspect circRBM33's cellular location. As depicted in Figure 3A, circRBM33 stayed in the cytoplasm at large in PC3 and C4-2, with 18S as the cytoplasmic indicator. Furthermore, the nuclear and cytoplasmic extraction assays yielded results consistent with FISH findings: circRBM33 was found in the cytoplasm (Figure 3B). Concerning the predominance of the ceRNA (competing endogenous RNA) mechanism in noncoding RNAs (ncRNAs) in the cytoplasm, we conducted an AGO2-RIP experiment to reveal whether circRBM33 functions in a ceRNA manner (Figure S3A). Unfortunately, the anti-AGO2 antibody had no interaction with circRBM33, ruling out circRBM33's claim to be a ceRNA. We then wondered whether circRBM33 has the potential to encode protein (Figure S3B). According to the results searched in circRNAdb, a circRNA database, circRBM33 was predicted to have a low possibility of encoding protein. Subsequently, we turned to the RNA-binding proteins (RBPs) that interact with circRBM33 for further exploration. We utilized two bioinformatics databases, catRAPID and CircInteractome, to explore its potent RBPs, and we harvested 10 candidates from CircInteractome (Figure S3C) and 19 from catRAPID omics (Figure S3D). With information integration from the two databases, FMR1, also known as FMRP (Fragile X Mental Retardation Protein), received the highest credit for interacting with circRBM33 (Figure 3C). More importantly, the FISH assays revealed their cytoplasmic colocalization, suggesting that an interaction between them may exist (Figure S3E). To further assess whether FMR1 interacts with circRBM33, we designed a probe targeting circRBM33 for ChIRP assays to lock down the molecules that interact with circRBM33. As presented in Figure 3D, qRT‒PCR confirmed that the RNA isolated from the ChIRP samples was circRBM33. Next, we implemented pull-down assays with proteins isolated from the ChIRP samples to examine whether FMR1 is one of the RBPs of circRBM33 (Figure 3E). In parallel, we conducted RIP assays with the anti-FMR1 antibody to further confirm the molecular binding between FMR1 and circRBM33 (Figure 3F). Furthermore, we uploaded the information of circRBM33 on a website called SRAMP, a sequence-based m6A modification site predictor, to investigate its potential m6A-modified sites (Figure 3G). All seven sites lie in Exon 2 and Exon 5. Consistently, the motif analysis above (Figure 1C) confirmed the authenticity of the prediction, indicating that circRBM33 is surely m6A-modified. Then, we asked whether circRBM33 interacts with FMR1 in a m6A-dependent manner. Since METTL3 is one of the most essential writers in m6A modification, we silenced METTL3 to alter the m6A level and to further assess our speculation (Figure 3H). Next, we performed MeRIP assays to examine whether METTL3 knockdown attenuates circRBM33 m6A modification. M1 and M2 are two circRBM33 segments that are amplified by the primers to represent the m6A levels (Figure 3G). Consequently, the MeRIP samples did not contain either M1 or M2 when METTL3 was downregulated, indicating that METTL3 participated in the m6A modification of circRBM33 (Figure 3I). Following this finding, we carried out FMR1-RIP assays to determine whether METTL3 engages in the FMR1-circRBM33 interaction (Figure 3J). Accordingly, suppressing METTL3 compromised the binding between FMR1 and circRBM33. Moreover, we applied a ChIRP assay to verify the indispensable role of m6A in the circRBM33 and FMR1 interaction, and the results indicated that knockdown of METTL3 remarkably impeded the circRBM33 pulldown of FMR1 (Figure 3K-L). Taken together, circRBM33 interacts with FMR1 in a m6A-dependent manner.

### FMR1 Promotes PCa Aggressiveness and is Related to a Poor Prognosis in PCa

**Figure 4:**
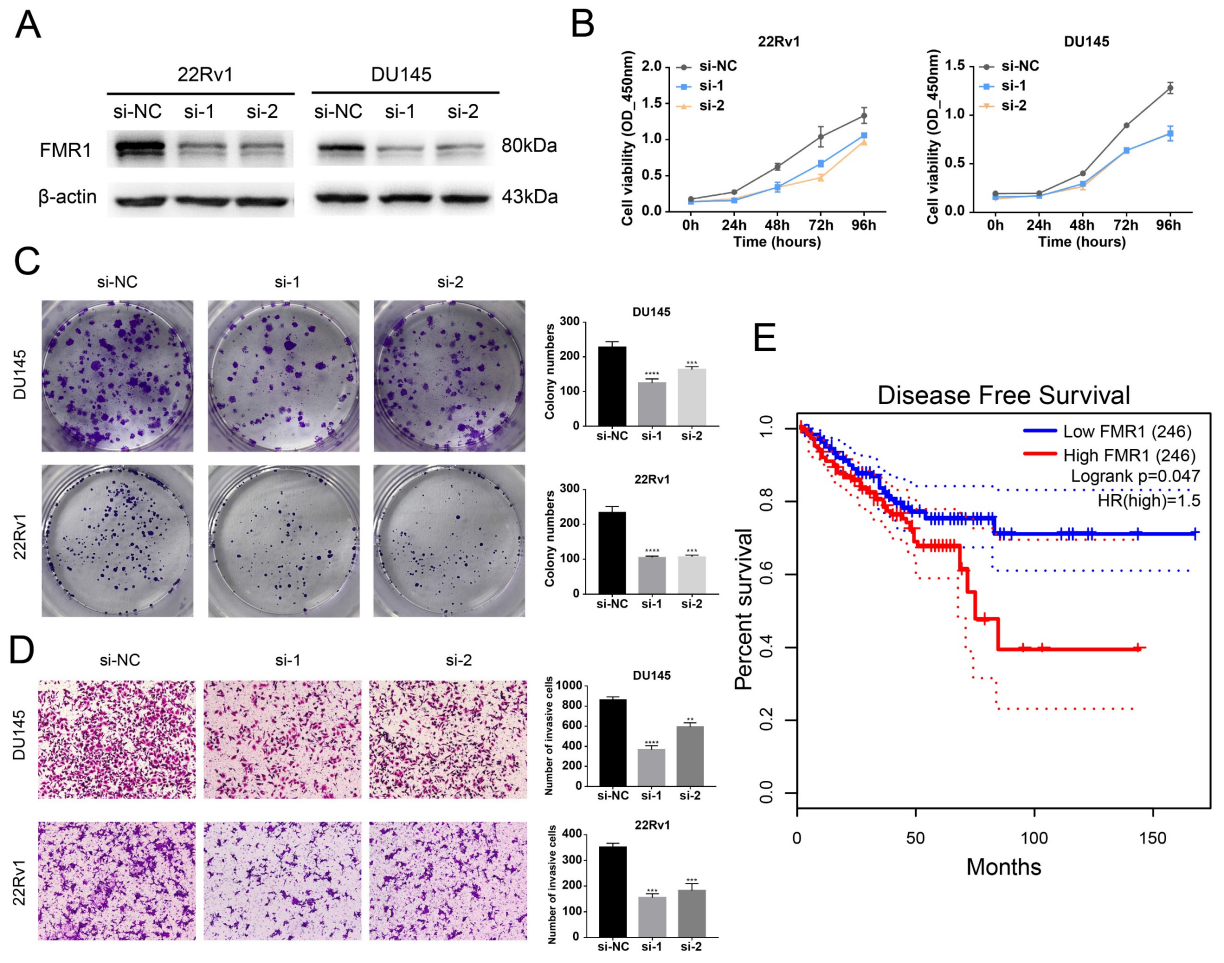
FMR1 promotes PCa aggressiveness and relates to a poor prognosis in PCa. (A) WB confirms the transfection efficiency of siRNA targeting FMR1 in 22Rv1 and DU145 cell lines. (B) CCK-8 assays demonstrate the proliferation changes after FMR1 downregulation in 22Rv1 and DU145 cell lines. (C) Plate colony formation assays exhibit FMR1-silenced PCa cells' ability to form colonies. (D) Transwell experiments determine invasiveness differences in FMR1-silenced PCa cells with si-NC as a negative control. (E) KM survival analysis validated FMR1's prognostic value in PCa.

After we validated that FMR1 interacts with circRBM33, we wondered whether FMR1 affects PCa. We first detected the basal expression of FMR1 in various PCa cell lines and found that FMR1 expression was relatively higher in 22Rv1 and Du145 cells and lower in C4-2 and PC-3 cells (Figure S4A). Then, we constructed FMR1-silenced cells via siRNA transfection in 22Rv1 and DU145 cells (Figure 4A). The 4-day cell viability measurement showed that suppressing FMR1 hampered PCa cell proliferation (Figure 4B). In line with this, the colony formation ability of PCa cells correspondingly declined after silencing FMR1 (Figure 4C). On the other hand, transwell assays demonstrated that downregulating FMR1 inhibited PCa cell invasiveness (Figure 4D). In short, FMR1 promotes PCa aggressiveness. Moreover, we examined the role of FMR1 in PCa prognosis (Figure 4E). Given the results from GEPIA2, patients with low levels of FMR1 yielded a better DFS than those with high FMR1 levels, indicating that FMR1 is related to a poor prognosis in PCa. In light of both circRBM33 and FMR1 acting as pro-tumor factors, we tested whether they affect each other at the transcriptional or translational level. First, WB assays demonstrated that neither upregulating nor silencing circRBM33 influenced FMR1 expression (Figure S4B-C). Second, we confirmed whether altering FMR1 affects circRBM33 (Figure S4D-E). qRT‒PCR assays showed that circRBM33 expression did not change much after overexpressing FMR1 and changed slightly when FMR1 was silenced (Figure S4F-G). Simultaneously, actinomycin D assays revealed no significant variation in the stability of circRBM33 after the overexpression or knockdown of FMR1 (Figure S4H). Altogether, circRBM33 did not regulate FMR1 expression in PCa, and vice versa. However, we determined a clinical correlation between FMR1 and circRBM33 in PCa via TMA (tissue microarray) (Figure S5). As shown in Figure S5A and Table 1, circRBM33 levels in tumour tissues were significantly higher than those in normal tissues, and the higher the GS was, the more circRBM33 was expressed. Likewise, IHC demonstrated that tumour tissues had higher FMR1 levels than normal tissues, and the worse the malignance was, the more FMR1 was expressed (Figure S5B & Table 1). Moreover, FMR1 scores varied between the low circRBM33-expression and high circRBM33-expression groups (Figure S5C). Tumour tissues in the high circRBM33-expression group tended to have higher FMR1 scores than those in the low circRBM33-expression group. Accordingly, the chi-square test confirmed the positive correlation between circRBM33 and FMR1 in PCa (Figure S5D). Taken together, we assume that circRBM33 interacts with FMR1 to form a complex that exerts its tumour-promoting effects in PCa.

### CircRBM33 Significantly Increases Mitochondrial Respiration in PCa Cells

**Figure 5:**
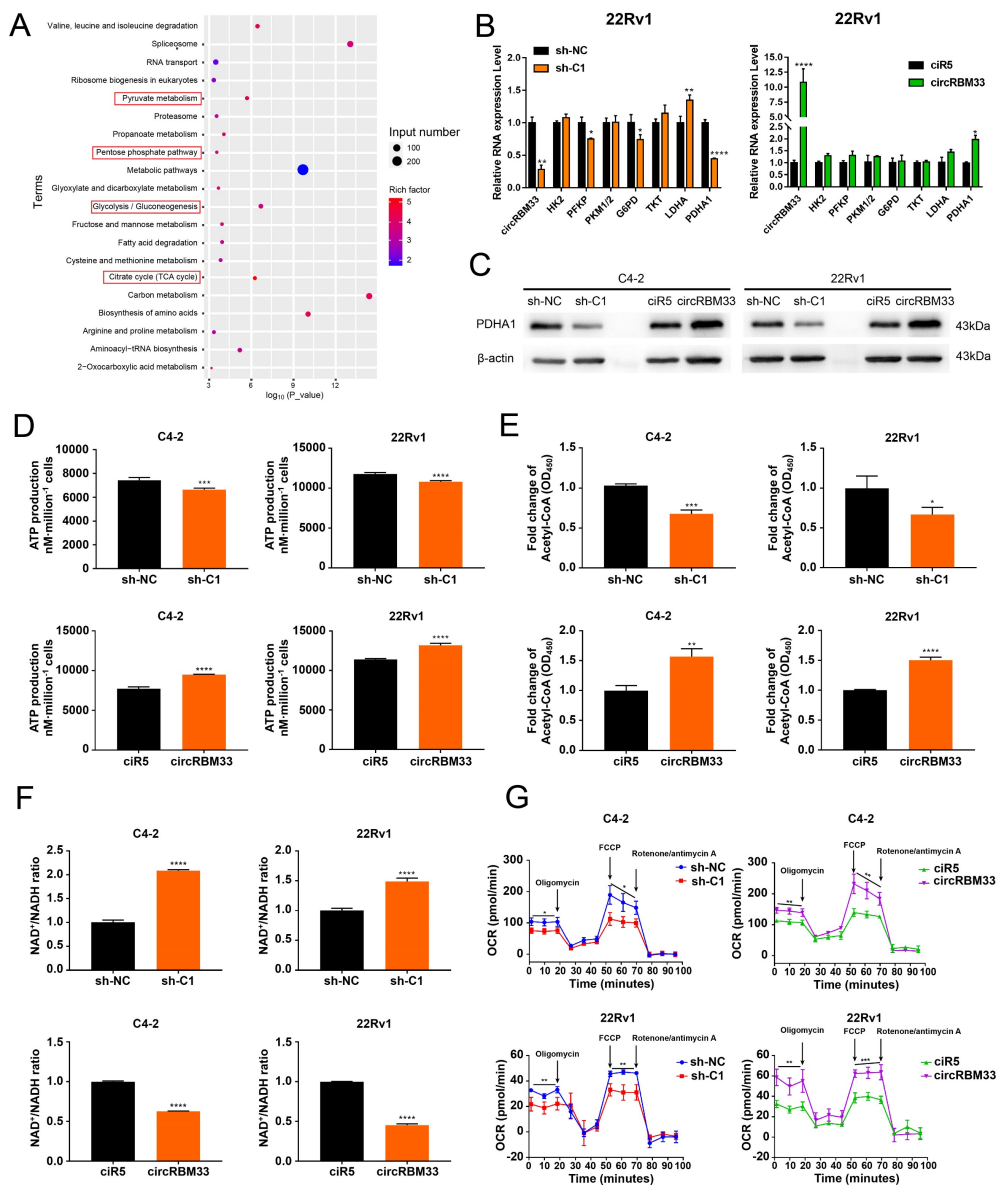
CircRBM33 significantly increases mitochondrial respiration in PCa cells. (A) KEGG pathway analysis of FMR1-RIP sequencing. (B) qRT-PCR determined the RNA expression levels of the key molecules involved in pyruvate metabolism, the pentose phosphate pathway, glycolysis/gluconeogenesis, and the citrate cycle in PCa cells when circRBM33 was upregulated or downregulated. (C) WB detects the PDHA1 expression differences in PCa cells after circRBM33 is overexpressed or silenced. (D-E) Detection of the changes in ATP production and acetyl-CoA levels in circRBM33-silenced and circRBM33-overexpressed cells compared to negative control cells. (F) Detection of the variation in the NAD+/NADH ratio in circRBM33-silenced and circRBM33-overexpressed cells compared to their own negative control cells. (G) Seahorse experiments detect the OCR in circRBM33-silenced and circRBM33-overexpressed cells in comparison to their own negative control cells.

With the speculation of a circRBM33-FMR1 functional complex, we performed a RIP experiment with an anti-FMR1 antibody, followed by FMR1-RIP sequencing to identify molecules associated with FMR1 (Table S4). Then, we subjected these molecules to functional enrichment analysis for further investigation (Figure 5A, Table S4). The findings indicate that the circRBM33/FMR1 complex may be engaged in four major biological processes: pyruvate metabolism, the pentose phosphate pathway, glycolysis/gluconeogenesis, and the citrate cycle. Hence, we monitored whether the activities of key molecules involved in the processes above changed at both the RNA and protein levels. The RNA expression of PFKP, LDHA, and PDHA1 decreased significantly in the circRBM33-downregulated 22Rv1 and C4-2 cell lines (Figure 5B & S6A). Notably, the protein expression of all these metabolism-related molecules, except PDHA1, exhibited no relativity to circRBM33 (Figure S6B). Nevertheless, the expression of PDHA1 at both the RNA and protein levels was positively related to that of circRBM33 (Figure 5B-C & S6A). PDHA1 represents the primary link between glycolysis and the tricarboxylic acid (TCA) cycle. Thus, circRBM33 likely participates in mitochondrial respiration considering the findings above. The expression of circRBM33 contributed to ATP production variation (Figure 5D). The circRBM33-overexpressing PCa cells exhibited enhanced ATP production, while the circRBM33-silenced cells displayed reduced ATP production. Consistent with this result, circRBM33 shared a positive correlation with acetyl-CoA but a negative correlation with the NAD+/NADH ratio, suggesting that circRBM33 boosts mitochondrial respiration (Figure 5E-F). Then, we further examined the mitochondrial respiratory capacity of PCa cells with different expression levels of circRBM33. In C4-2 and 22Rv1 cells, the basal and maximal respiratory capacities of PCa cells with high circRBM33 expression were higher than those of PCa cells with low circRBM33 expression (Figure 5G). These results suggest that circRBM33 boosts mitochondrial respiration for PCa cell growth and aggressiveness.

### Downregulating FMR1 Reduces circRBM33-mediated Aggressive Phenotypes in PCa Cells

**Figure 6:**
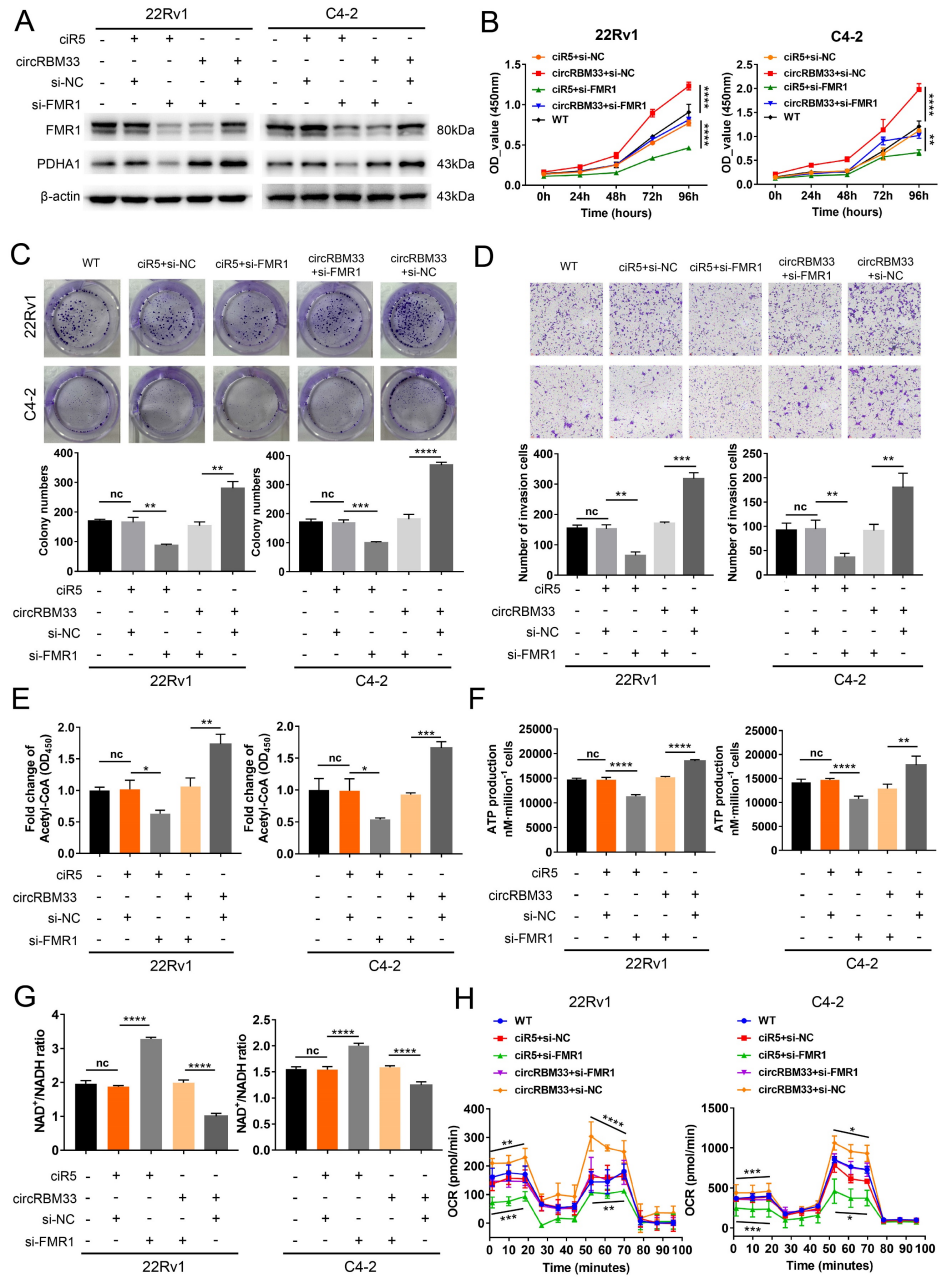
Downregulating FMR1 impairs circRBM33-mediated aggressive phenotypes in PCa cells. (A) WB validates the influence of FMR1 knockdown on PDHA expression in circRBM33-overexpressed cells (22Rv1 and C4-2). (B) The CCK-8 assay examines the influence of FMR1 silencing on cell growth in circRBM33-overexpressed cells. (C) The influence of FMR1 downregulation on colony formation ability was examined in circRBM33-overexpressed cells. (D) The Transwell assay determines the impact of FMR1 silencing on invasiveness in circRBM33-overexpressed cells. (E) Detection of the NAD^+^/NADH ratio changes in the circRBM33-overexpressed cells when FMR1 is downregulated. (F-G) Detection of ATP and acetyl-CoA production changes in circRBM33-overexpressed cells when FMR1 is downregulated. (H) Seahorse experiments detects the basal and maximal OCR in the circRBM33-overexpressed cells when FMR1 was downregulated.

We knocked down FMR1 using siRNA in circRBM33-overexpressing PCa cells to investigate the role of FMR1 in circRBM33-mediated aggressive phenotypes (Figure 6A). We first examined whether repressing FMR1 expression influences circRBM33-mediated enhanced PCa cell growth. As expected, knockdown of FMR1 partially attenuated cell proliferation in 22Rv1 and C4-2 cells (Figure 6B). In line with this, plate colony formation assays confirmed similar results (Figure 6C).

FMR1 downregulation jeopardized the invasiveness of circRBM33-overexpressing PCa cells (Figure 6D). Overall, these findings reveal that silencing FMR1 partially inhibits circRBM33-mediated aggressiveness in PCa cells. We then investigated whether FMR1 interacts with circRBM33 in the regulation of mitochondrial respiration. WB assays showed that PDHA1 expression decreased when FMR1 was silenced in circRBM33-upregulated 22Rv1 and C4-2 cells (Figure 6A), preliminarily confirming that FMR1 participates in regulating circRBM33-mediated mitochondrial respiration. We next measured the FMR1-circRBM33 interaction. When FMR1 was downregulated, we detected an increase in the NAD^+^/NADH ratio but a decrease in ATP production and acetyl-CoA in circRBM33-overexpressing 22Rv1 and C4-2 cells (Figure 6E-G), indicating that FMR1 coregulates mitochondrial respiration with circRBM33. Consistent with the results above, the basal and maximal respiratory capacities of circRBM33-upregulated PCa cells dropped when FMR1 expression was suppressed (Figure 6H). These data clearly demonstrated that knockdown of FMR1 reduces circRBM33-mediated aggressiveness in PCa cells via the FMR1-circRBM33 interaction in mitochondrial respiration. Furthermore, to determine whether circRBM33 or FMR1 exhibits oncogenic effects on the normal prostate cell line RWPE-1, we overexpressed circRBM33 and FMR1 in RWPE-1 cells (Fig. S7A-B) and performed functional assays. Unexpectedly, neither overexpression of circRBM33 nor FMR1 affected the proliferation or migration ability of RWPE-1 cells (Figure S7C-D).

### The circRBM33-FMR1 Complex Sustained PDHA1 mRNA Stability

**Figure 7:**
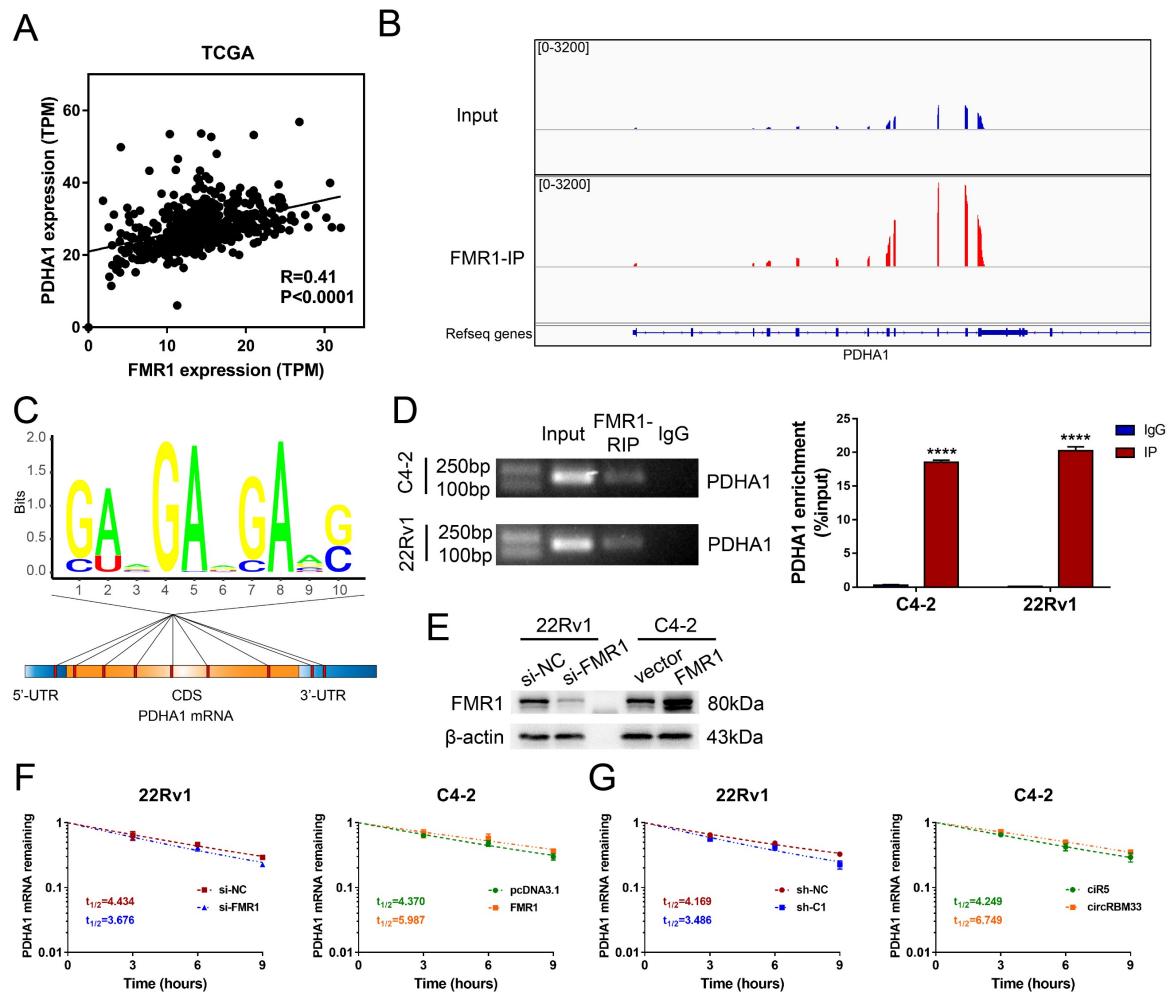
CircRBM33-FMR1 Complex sustains PDHA1 mRNA stability. (A) The PDHA1 and FMR1 expression correlation analysis in TCGA database. (B) Using the Integrative Genomics Viewer (IGV) visualizes the FMR1 binding regions of PDHA1. (C) The distribution of the potential FMR1 binding sites in PDHA1. (D) FMR1-RIP assays confirm the enrichments of PDHA1. (E) WB confirmed the transfection efficiency of downregulating or overexpressing FMR1 in PCa cell lines. (F) The variation in PDHA1 stability when FMR1 is upregulated or downregulated. (G) Actinomycin D assay detects the mRNA stability of PDHA1 when circRBM33 is upregulated or downregulated.

Our findings revealed that both circRBM33 and FMR1 regulate PDHA1, and circRBM33 hypothetically interacts with FMR1 to function as a whole. We then examined whether there is an expression correlation between PDHA1 and FMR1. As suspected, PDHA1 shared a positive correlation with FMR1 (Figure 7A, R = 0.41, p < 0.0001). We then investigated the gene sequencing data of FMR1-IP. Surprisingly, the PDHA1 peaks resembled those of FMR1-IP to some extent (Figure 7B). Furthermore, motif analysis demonstrated that several potential m6A-modified sites lie in PDHA1 mRNA, including its 5'-UTR, CDS, and 3'-UTR regions, suggesting that the circRBM33-FMR1 complex regulates PDHA1 by binding these sites (Figure 7C). To assess whether FMR1 participates in PDHA1 regulation, we confirmed an interaction between these two molecules via RIP assays. In 22Rv1 and C4-2 cells, PDHA1 mRNA was enriched significantly by RIP assay using anti-FMR1 antibody (Figure 7D). Given that FMR1 was reported to be involved in the modulation of targeted mRNA stability or translation 30, we further defined how exactly the circRBM33-FMR1 complex regulates PDHA1. After validating the transfection efficiency of knockdown or overexpression of FMR1, we performed PDHA1 mRNA stability assays (Figure 7E). As a result, silencing FMR1 decreased the half-life of PDHA1 mRNA, whereas overexpressing FMR1 prolonged the half-life of PDHA1 mRNA (Figure 7F). Likewise, we observed similar results when upregulating or downregulating circRBM33 (Figure 7G). Taken together, the circRBM33-FMR1 complex sustains PDHA1 mRNA stability in a m6A-dependent manner.

### CircRBM33 Promotes PCa Tumour Growth *in vivo*

**Figure 8:**
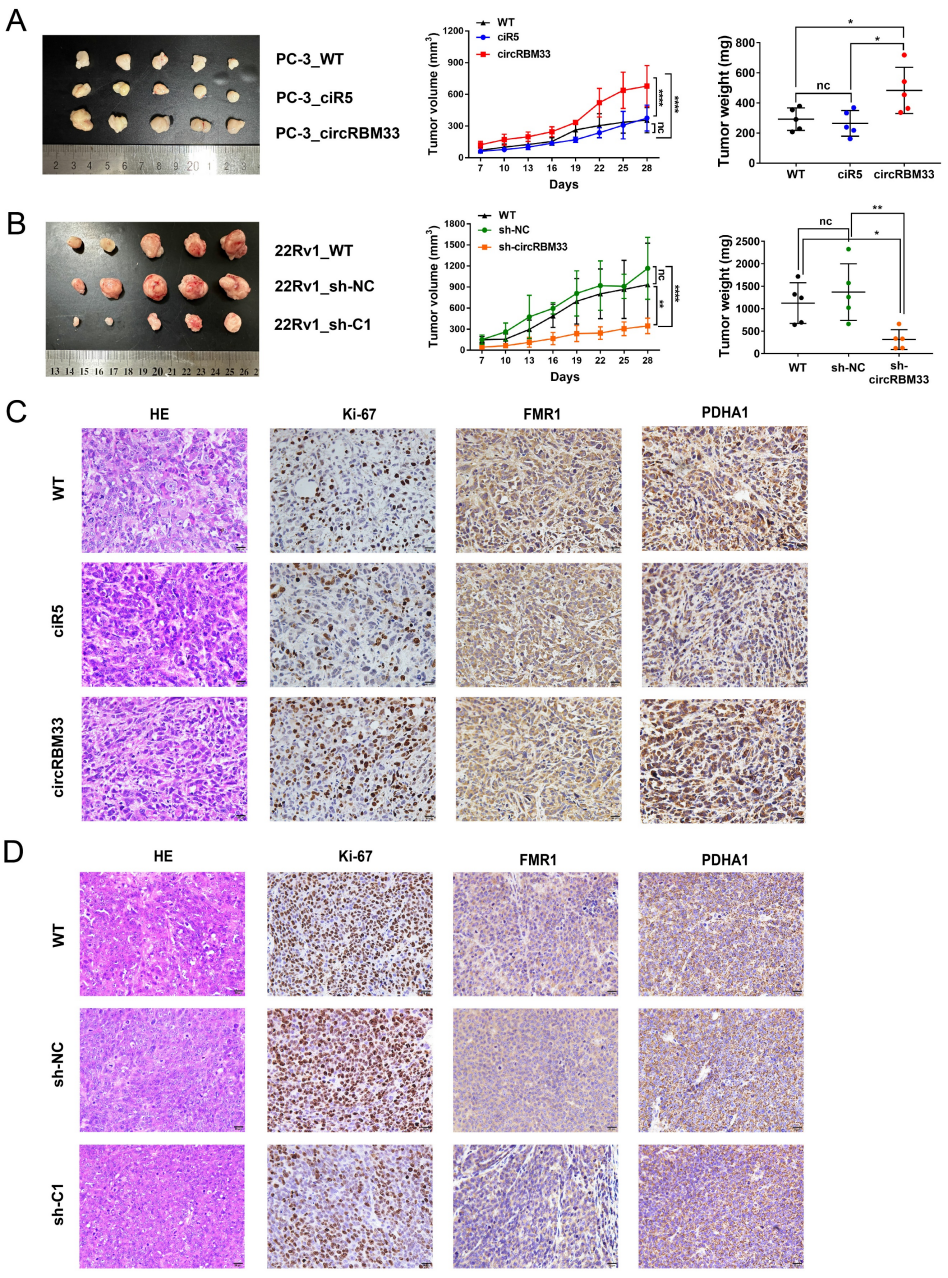
CircRBM33 promotes the PCa tumor growth *in vivo*. (A) The influence of overexpressing circRBM33 on subcutaneous PCa tumor growth in nude mice. (B) The impact of circRBM33 knockdown on subcutaneous PCa tumor growth in nude mice. (C) The IHC assay detects the expression changes in Ki67, FMR1 and PDHA1 in circRBM33-overexpressed PCa subcutaneous tumors. (D) The IHC assay detects the expression changes in Ki67, FMR1 and PDHA1 in circRBM33-silenced PCa subcutaneous tumors.

To scrutinize the role of circRBM33 in PCa *in vivo*, we subcutaneously injected circRBM33-upregulated or circRBM33-silenced PCa cells into BALB/c nude mice to induce xenograft tumours. At first, tumour volume was measured once every three days after Day 7, and by the end of Day 28, all mice were sacrificed for dissection and tumour measurement. As shown in Figure 8A, upregulation of circRBM33 boosted tumour growth in volume and weight, both visually and statistically. The knockdown of circRBM33 hindered tumour progression in the opposite direction (Figure 8B). We then extracted the xenograft tumours for HE staining and immunohistochemistry assays. As expected, Ki67 and PDHA1 were expressed more strongly in the circRBM33-overexpressing tumour tissues than in the negative control tumours, while FMR1 was slightly altered (Figure 8C). In contrast, both Ki67 and PDHA1 were reduced when circRBM33 was silenced (Figure 8D). Overall, circRBM33 promotes PCa tumour growth *in vivo*.

### Knockdown of circRBM33 Enhances PCa Cell Responses to ARSI Therapy *in vitro* and *in vivo*

**Figure 9:**
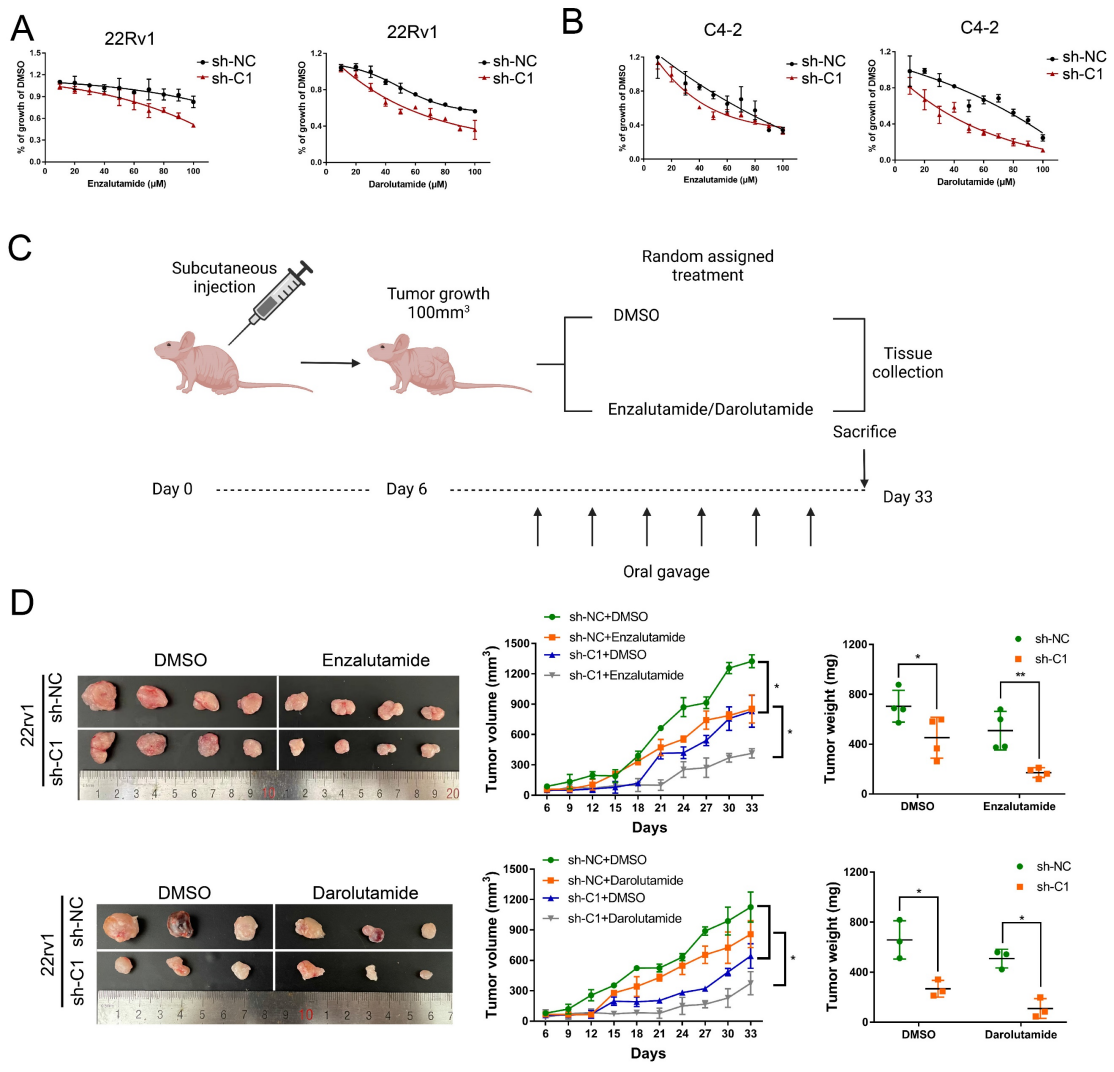
Knockdown of circRBM33 enhances tumor sensitivity to ARSI therapy in PCa. (A) The effects of ARSIs (Enzalutamide and Darolutamide) on circRBM33-depleted 22Rv1 cells in comparison to the negative control. (B) The effects of ARSIs (Enzalutamide and Darolutamide) on circRBM33-silenced C4-2 cells in comparison to the negative control. (C) The workflow shows the subcutaneous tumor mouse model construction based on ARSIs (enzalutamide and darolutamide) oral administration. (D) The comparison of ARSIs' influence on the growth of circRBM33-downregulated tumors.

CRPC cells employ greater mitochondrial metabolism, resulting in increased oxidative phosphorylation and leaving them vulnerable to mitochondrial metabolism-focused therapy, as mentioned in a previous article 29. Given that circRBM33 has an indirect impact on mitochondrial respiration, we finally assessed whether silencing circRBM33 influences PCa cell sensitivity to ARSIs (androgen receptor signalling inhibitors). To evaluate PCa cell responses to ARSIs, we employed two ARSIs, enzalutamide and darolutamide, in cell culture (Figure 9A-B). As the administrative concentration increased, the 22Rv1 and C4-2 cells exhibited declining curves in cell growth. In contrast, restrained cell growth was more apparent in circRBM33-downregulated cells.

Then, we subcutaneously injected circRBM33-silenced cells (sh-C1) and negative control cells (sh-NC) into BALB/c nude mice to induce xenograft tumours. Simultaneously, we separated these mice into two subgroups for random oral administration of either enzalutamide/darolutamide or DMSO (Figure 9C). We monitored the tumour size for over a month and then measured the tumour weight after sacrificing these mice. As a result, circRBM33-silenced tumours exhibited more restrictive growth in volume and weight than control tumours. In parallel, ARSI administration enhanced growth restriction compared to DMSO administration (Figure 9D). Since circRBM33 is included in the sensitivity of ARSIs in PCa, we asked whether circRBM33 or FMR1 is involved in the regulation of the AR signalling pathway. We applied Pearson correlation between circRBM33 or FMR1 and AR expression in GSE113124 data and found that both correlations were relatively low (Figure S8A-B). Next, we performed qRT‒PCR and WB to detect the expression change of AR and AR-V7 (naturally expressed in 22Rv1 and leads to resistance to enzalutamide) by manipulating circRBM33 and FMR1 expression in 22Rv1 cells. Downregulation or overexpression of circRBM33 or FMR1 decreased or increased the expression of AR-V7 instead of AR, respectively (Figure S8C-F). However, knockdown or upregulation of circRBM33 or FMR1 did not reduce or increase the stability of AR-V7 mRNA, respectively (Figure S8G). In conclusion, the knockdown of circRBM33 improves PCa cell responses to ARSI therapy *in vitro* and *in vivo* partially by regulating AR-V7 expression.

## Discussion

Prostate cancer, which is the most threatening cancer in American males, is a hormone-sensitive tumour in the primary stage 1. However, nearly all PCa patients will progress to CRPC after standard systemic ADT 4. The median survival time of metastatic CRPC patients is approximately 12 months in the natural course, and there is currently no curative medicine 5. Thus, searching for new therapeutic targets for PCa is worthy of further study. In addition to genetic mutation or transcript variants of AR or other signalling pathway activation, epigenetic change is also important in driving PCa progression 7, 31. As a new epigenetic modification, RNA m6A can be referred to epitranscriptomic modulation, which extensively exists in various types of RNA, including mRNA and noncoding RNA 17. Herein, we focused on the relationship between m6A modification and circRNAs and investigated their functional role in PCa progression.

Numerous studies have reported that m6A modification participates in the biogenesis and diverse functions of circRNAs. For example, Liu et al. found that METTL3 mediates the m6A modification of circIGF2BP3 and confers circularization 32. Additionally, m6A-modified circNSUN2 can be recognized by YTHDC1 and exported from the nucleus to the cytoplasm 33. Moreover, some specific m6A readers, such as IGF2BP2, could interact with circRNA to enhance its stability 34, 35. In our study, we validated that circRBM33 was methylated by METTL3 and then interacted with FMR1 in a m6A manner. However, FMR1 presented no effect on the stability of circRNA but formed a binary complex with circRBM33 to regulate downstream targets. Substantial studies have demonstrated that the absence of FMR1 protein is closely related to fragile X syndrome, including mental retardation and abnormal behaviour. Silencing of FMR1 was attributed to expanded CCG repeats and subsequent methylation of its promoter region 36. Emerging evidence has shown that FMR1 plays an important role in tumorigenesis and metastasis. For instance, Chen's study indicated that the long noncoding (lncRNA) RNA BC200 acted as a scaffold of FMR1 to coregulate the stability of its targeted mRNA HNF4α. Conversely, HNF4α transactivated lncRNA BC200 to form a positive feedback loop, thereby promoting tumour growth and metastasis in invasive mucinous lung adenocarcinoma 37. Additionally, Carotti et al. also found that FMR1 was upregulated in intrahepatic cholangiocarcinoma, bound to Cortactin (a marker of mature invadopodia) mRNA to mediate its expression, and then promoted cell migration and invasiveness by regulating invadopodia formation 38. Consistently, our results showed that silencing of FMR1 inhibited cell growth and invasion in PCa cells as well. Clinically, high expression of FMR1 predicts short progression-free survival in patients with PCa. More importantly, the protein expression level of FMR1 was positively correlated with the expression level of circRBM33 in our tissue microarrays, which further suggested that the circRBM33-FMR1 binary complex might coactivate tumour progression in PCa. In addition, we confirmed through functional experiments that circRBM33/FMR11 has no tumour-promoting effect on the normal prostate cell line RWPE-1. We hypothesize that inactivation of various repressor genes or hyperactivation of oncogenes leads to tumour-like behaviour in cancer cells, whereas chromatin accessibility or the transcriptional state of genes remains stable or maintains homeostasis in normal cells 4. Thus, just one or two changes in gene expression had little effect on normal cells. Meanwhile, this may further explain the important role of circRBM33/FMR1 in PCa progression rather than malignant transformation of normal cells.

It has been reported that FMR1, a classic RNA binding protein, plays an important role in mRNA stability and translation modulation 30, 39. Then, we performed anti-FMR1-RIP sequencing to screen the FMR1-regulating targets. In our KEGG enrichment analysis among all FMR1-binding mRNAs, we observed that FMR1-targeting mRNAs were mainly enriched in glycolysis, the pentose phosphate pathway, and the oxidative phosphorylation process. Considering that FMR1 not only influences the stability of mRNA but also modulates the translation of some targeted mRNAs, we started to examine the expression variation of those key enzymes when upregulating or downregulating circRBM33 at both the RNA and protein levels. Apparently, circBRM33 knockdown significantly increased or decreased the expression of PDHA1. PDHA1 is a main unit of the pyruvate dehydrogenase complex, which is responsible for the conversion of pyruvate to acetyl-CoA in mitochondria 40. PDHA1 seems to have a paradoxical role in regulating tumour growth and metastasis. It has been reported that overexpression of PDHA1 can inhibit aerobic glycolysis and result in mitochondria-mediated apoptosis in hepatocellular cancer 41. Additionally, other surveys elucidated that PDHA1 might play an antitumour role in cancer and that low expression of PDHA1 predicted a poor outcome in these tumours 42, 43. However, compared to Warburg effect-dependent cancer cells, several kinds of cancer rely on the TCA cycle as well. In cholangiocarcinoma, elevated PGC1α increased PDHA1 and mitochondrial pyruvate carrier 1 expression through the transcription program, thereby promoting cancer metastasis 44. Similarly, PDHA1 was also validated to promote PCa tumorigenesis by stimulating the TCA cycle and subsequently activating oxidative phosphorylation 45. Indeed, only modulating PDHA1 expression is not sufficient to explain how it drives the aggressiveness of cancer cells. To address this, this study provided valuable findings that PDHA1 can be transported from the cytoplasm to the nucleus in PCa cells and enhance lipogenesis-related gene transcription, thereby inducing lipid biosynthesis. More interestingly, prostate secretory epithelial cells seemed to hold an "incomplete" TCA cycle due to inhibition of aconitase, which is required for the synthesis and secretion of large amounts of citrate into semen 46-48. Thus, it is not surprising that PCa is more dependent on mitochondrial function than other kinds of tumours.

Our study revealed that the circRBM33/FMR1/PDHA1 regulatory axis was involved in strengthening the mitochondrial oxidative phosphorylation process in PCa cells, especially in castration-resistant cell lines, which was consistent with a previous report that the ox-pho pathway was enhanced in CRPC 29. It was confirmed that suppression of the ox-pho process using specific inhibitors significantly increased CRPC sensitivity to enzalutamide. To examine whether circRBM33 was involved in the ARSI therapy response, we applied two FDA-approved ARSIs, enzalutamide and darolutamide, to the treatment of PCa *in vitro* and *in vivo*. Surprisingly, knockdown of circRBM33 synergistically increased enzalutamide and darolutamide antitumour activity in PCa. Little direct evidence has confirmed the relationship between the ox-pho process and ARSI therapy response in PCa. Intratumoral androgen synthesis is one of the mechanisms resisting androgen/AR signalling pathway blockade in CRPC 49, 50. Most importantly, lipid/cholesterol is the precursor of synthesizing steroids, including androgen 49-51. Thus, nucleus-localized PDHA1-driven lipid/cholesterol biosynthesis might take part in ARSI sensitivity. In addition, we also revealed that downregulation of circRBM33/FMR1 suppressed AR-V7 expression in PCa, which could be another reason to explain why decreasing circRBM33 increased the sensitivity of PCa to ARSIs.

## Conclusion

**Figure 10:**
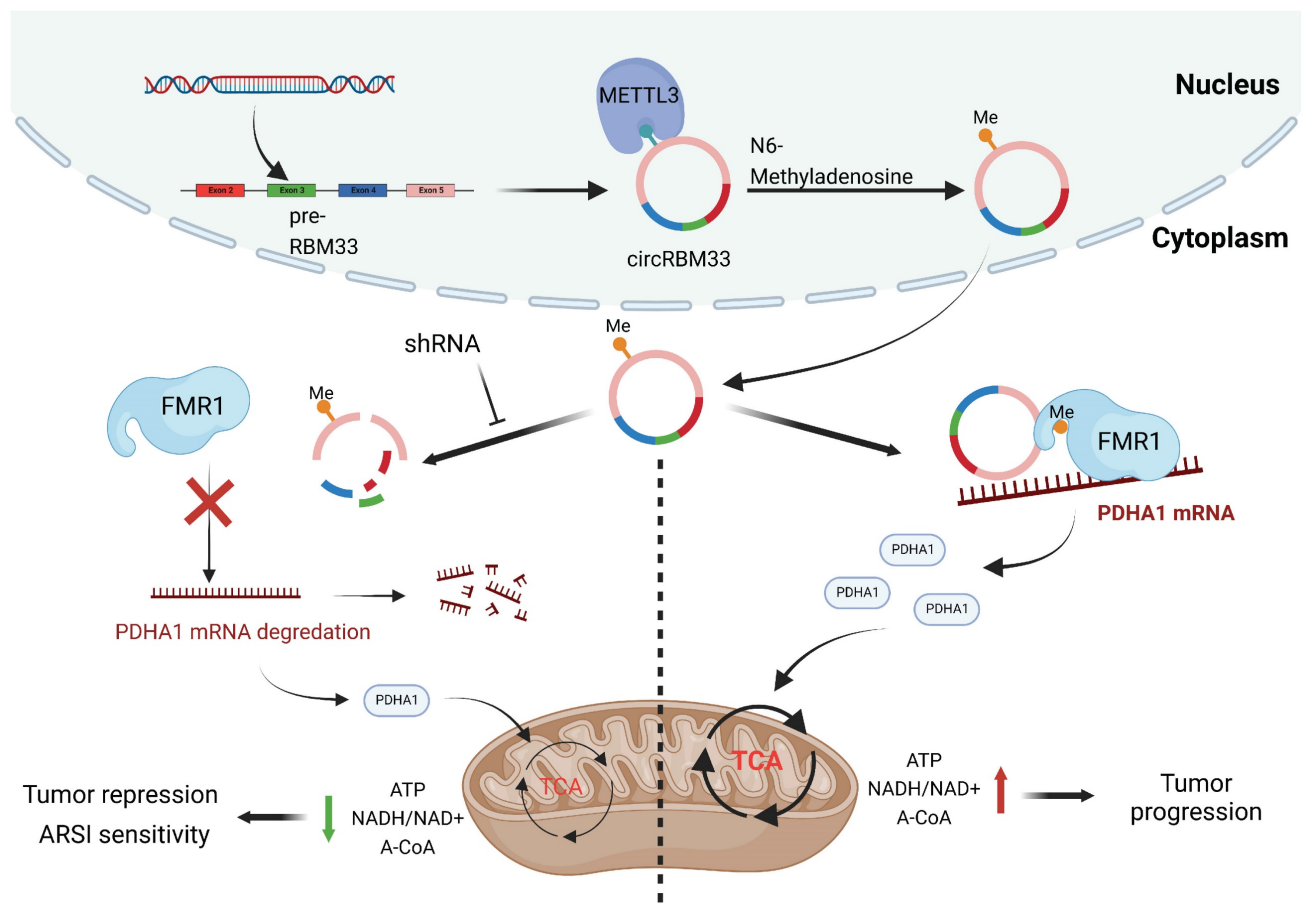
The schematic graph presents the potential molecular mechanism of circRBM33 regarding tumor aggressiveness modulation and ARSI therapy sensitivity in prostate cancer.

In summary, our findings revealed that circRBM33 was m6A-modified, promoted tumour growth and invasion, and correlated with poor prognosis in PCa. Mechanistically, circRBM33 interacted with FMR1 in a m6A manner to form a binary complex, which enhanced mitochondrial metabolism by regulating PDHA1. Moreover, suppressed circRBM33 increased ARSI antitumour activity in CRPC cell lines. Altogether, circRBM33 is a potential therapeutic target in PCa, especially in CRPC (Figure 10).

## Supplementary Material

Supplementary figures, table 3.

Supplementary table 1.

Supplementary table 2.

Supplementary table 4.

## Figures and Tables

**Table 1:** Clinicopathological characteristic of circRBM33/FMR1 in prostate cancer (TMA)

			N	circRBM33 expression		χ2	*p* value		N	FMR1 expression	*p* value
	Group			low	high					(Mean ± SD)	
Type	Normal		10	9	1	4.102	0.043*^a^*		10	3.500 ± 1.958	0.000*^c^*
	Cancerous		47	23	24				46	6.152 ± 0.842	
Gleason score	>7		16*	4	12	6.751	0.034*^b^*		16^#^	6.375 ± 0.806	0.001*^d^*
	=7		21	11	10				21	6.095 ± 0.768	
	<7		9	7	2				9	5.111 ± 0.782	
Age	<65		13	8	5	1.142	0.285*^b^*		34	5.941 ± 0.952	0.648*^c^*
	≥65		34	15	19				13	6.077 ± 0.760	

The expression of circRBM33 was determined by FISH: *^a^* represented *p* value was tested by chi-squared (χ^2^) test with Yates's correction for continuity;*^b^* represented *p* value was tested by Pearson's χ^2^ test. The expression of FMR1 was determined by IHC: ^c^ represented *p* value was tested by Student's *t* test; ^d^ represented *p* value was tested by One-way ANOVA test. * means the expression difference of circRBM33 between GS>7 and GS<7 was statistically different; ^#^ means the expression difference of FMR1 between GS>7 and GS<7 was statistically different.
