# Supplementary material for: M6A-modified circRBM33 promotes prostate cancer progression via PDHA1-mediated mitochondrial respiration regulation and presents a potential target for ARSI therapy
Source: Int J Biol Sci. 2023 Mar 5;19(5):1543–63. doi: 10.7150/ijbs.77133 (PMC10086746; doi:10.7150/ijbs.77133)
Supplement: Supplementary file 1 — Supplementary figures, table 3. [file ijbsv19p1543s1.pdf]

Supplementary Figure 1 The expression relationship between circRBM33 and linearRBM33 in PCa. (A) qRT-PCR confirms the transfection efficiency of circRBM33 knockdown or overexpression and the RNA expression change of linear RBM33 in different PCa cell lines. (B) WB shows the RBM33 protein expression levels in circRBM33-upregulated or downregulated PCa cells.

Supplementary Figure 2 Decreasing m6A level retarded circRBM33 induced pro-tumor effect. (A) Dot blot assays confirms the m6A level in C4-2 and PC-3 cell lines with or without gradient concentration of STM2457 treatment. (B) CCK-8 assays examines the cell viability in circRBM33-overexpressed PCa cells with or without STM2457 treatment. (C) Plate colony formation assays determine the colony formation ability of PCa cells after circRBM33 overexpression with or without STM2457 treatment. (D) Transwell assays confirm the invasiveness of circRBM33-overexpressed PCa cells with or without STM2457 treatment.

Supplementary Figure 3 CircRBM33 potentially interacts with FMR1. (A) AGO2-RIP experiments show the enrichment of circRBM33 by the AGO2 antibody. ciRS-7 acts as a positive control. (B) The exploration of circRBM33's protein coding potential using the circRNADb database.(C) The CircInteractome database was used to identify potential circRBM33 RNA-binding proteins.(D) The catRAPID database was used to identify potential circRBM33 RNA-binding proteins.(E) FISH and immunofluorescence (IF) experiments demonstrate the co-localization of circRBM33 and FMR1 in PCa cells.

Supplementary Figure 4 Exploring the relationship between circRBM33 and FMR1 in PCa. (A) WB detects the basal expression of FMR1 in different PCa cells. (B)&(C) FMR1 protein expression levels are detected by Western blotting in circRBM33-overexpressed and circRBM33-silenced states when compared to respective negative controls. (D)&(E) WB confirms the transfection efficiency of knockdown or overexpression of FMR1 in PCa cells. (F)&(G) qRT-PCR detects circRBM33 expression levels in FMR1-silenced or overexpressed PCa cells. (H) Actinomycin D assays validate circRBM33's stability in the FMR1-downregulated or upregulated state.

Supplementary Figure 5 Clinical correlation between FMR1 and circRBM33 in PCa. (A) FISH assays demonstrate the circRBM33 expression disparity between tumor tissues with different Gleason scores and benign prostate epithelial tissues. (B) IHC assays detect FMR1 expression differences between benign prostate epithelial tissues and tumoral prostate tissues with scores < 8 or > 8 in Gleason scores. (C) The statistics analysis of the FMR1 score difference between tumoral tissues with low circRBM33 expression and those with high circRBM33 expression. (D) The chi-square test confirms the expression correlation between circRBM33 and FMR1.

Supplementary Figure 6 The relation between circRBM33 and glycolysis-related pathway molecules. (A) qRT-PCR detects the RNA expression levels of HK2, PFKP, PKM1/2, G6PD, TKT, LDHA, and PDHA1 in C4-2 cells in circRBM33-upregulated or downregulated states. (B)WB determines the protein expression levels of HK2, PFKP, PKM1/2, G6PD, TKT,

LDHA, and PDHA1 in circRBM33-upregulated and circRBM33-downregulated or upregulated cells.

Supplementary Figure 7 Verification of the oncogenic role of circRBM33 or FMR1 in RWPE-1 cells. (A) qRT-PCR verifies the transfection efficiency of circRBM33 overexpression in RWPE-1 cells. (B) qRT-PCR and WB validates the transfection efficiency of FMR1 overexpression in RWPE-1 cells. (C) CCK-8 assays determine the cell viability of RPWE-1 cells after the upregulation of circRBM33 or FMR1. (D) Plate colony formation assay examine the colony formation capability of circRBM33 or FMR1 overexpressed RWPE-1 cells. (E) Transwell assays detects the migration ability of RWPE-1 cells when overexpressing circRBM33 or FMR1.

Supplementary Figure 8 Identification of the regulatory role of circRBM33 or FMR1 in AR pathway. (A)&(B) Pearson correlation between circRBM33 or FMR1 with AR in RNA level. (C)&(D) qRT-PCR validates the effects of circRBM33 or FMR1 on AR or AR-V7 mRNA expression in 22Rv1 cells. (E) WB examines AR or AR-V7 protein expression after the upregulation or downregulation of circRBM33 or FMR1 in 22Rv1 cells. (F) Actinomycin D experiments determine the mRNA stability of AR-V7 in circRBM33 or FMR1 overexpressed or knockdown 22Rv1 cell lines.

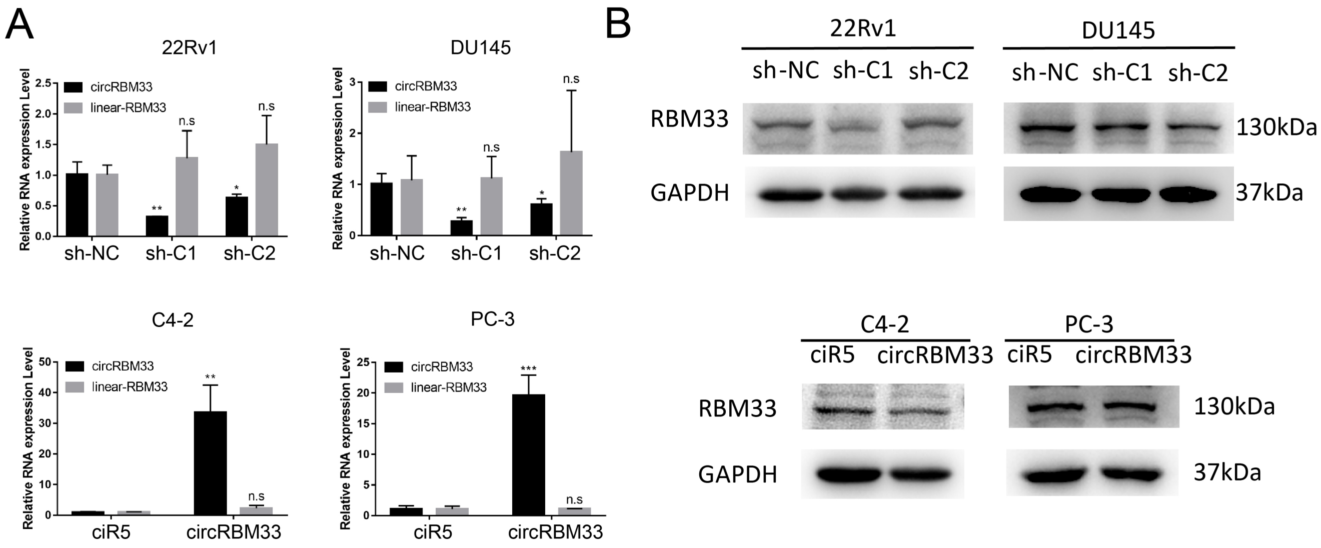

Supplementary Figure 1

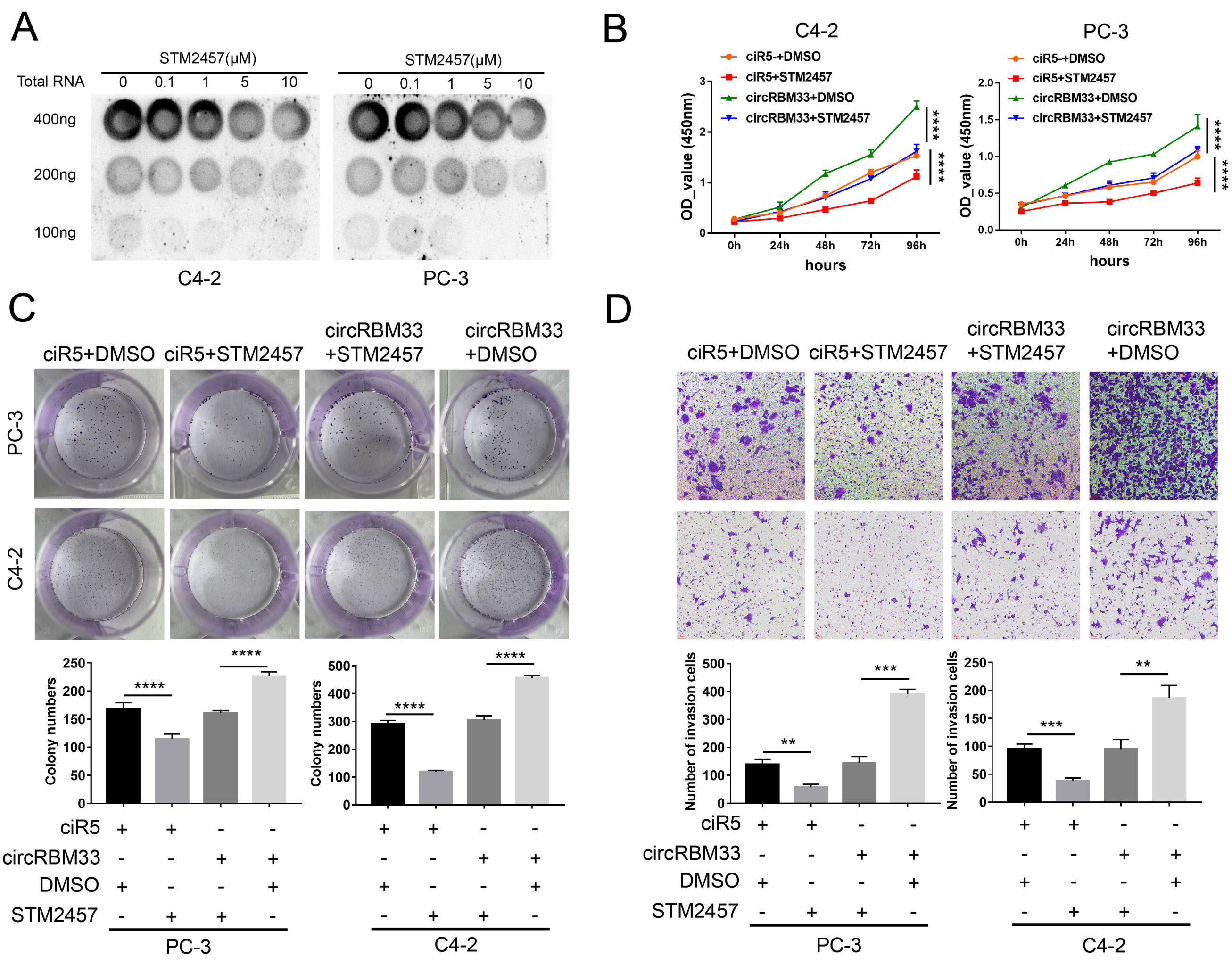

Supplementary Figure 2

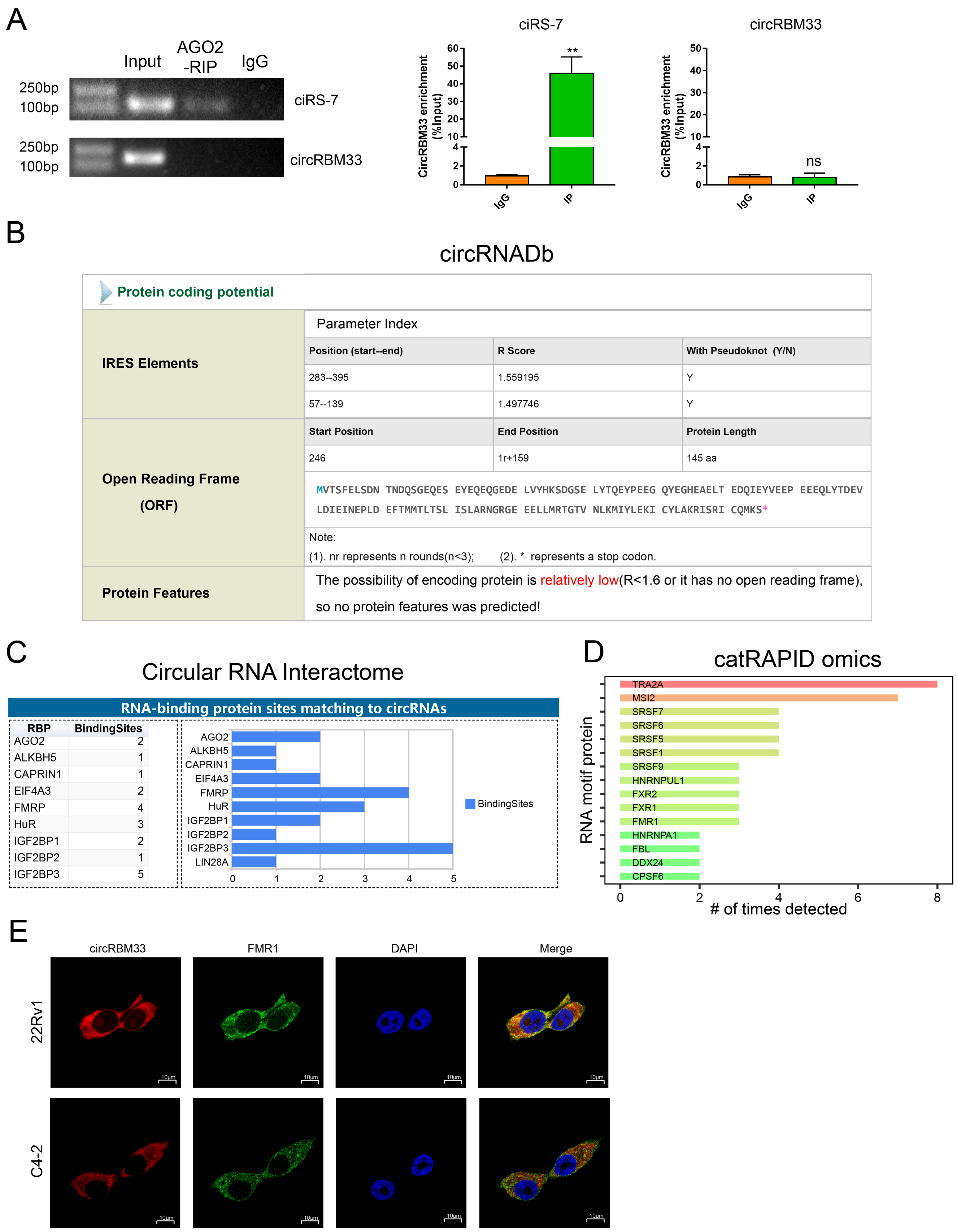

Supplementary Figure 3

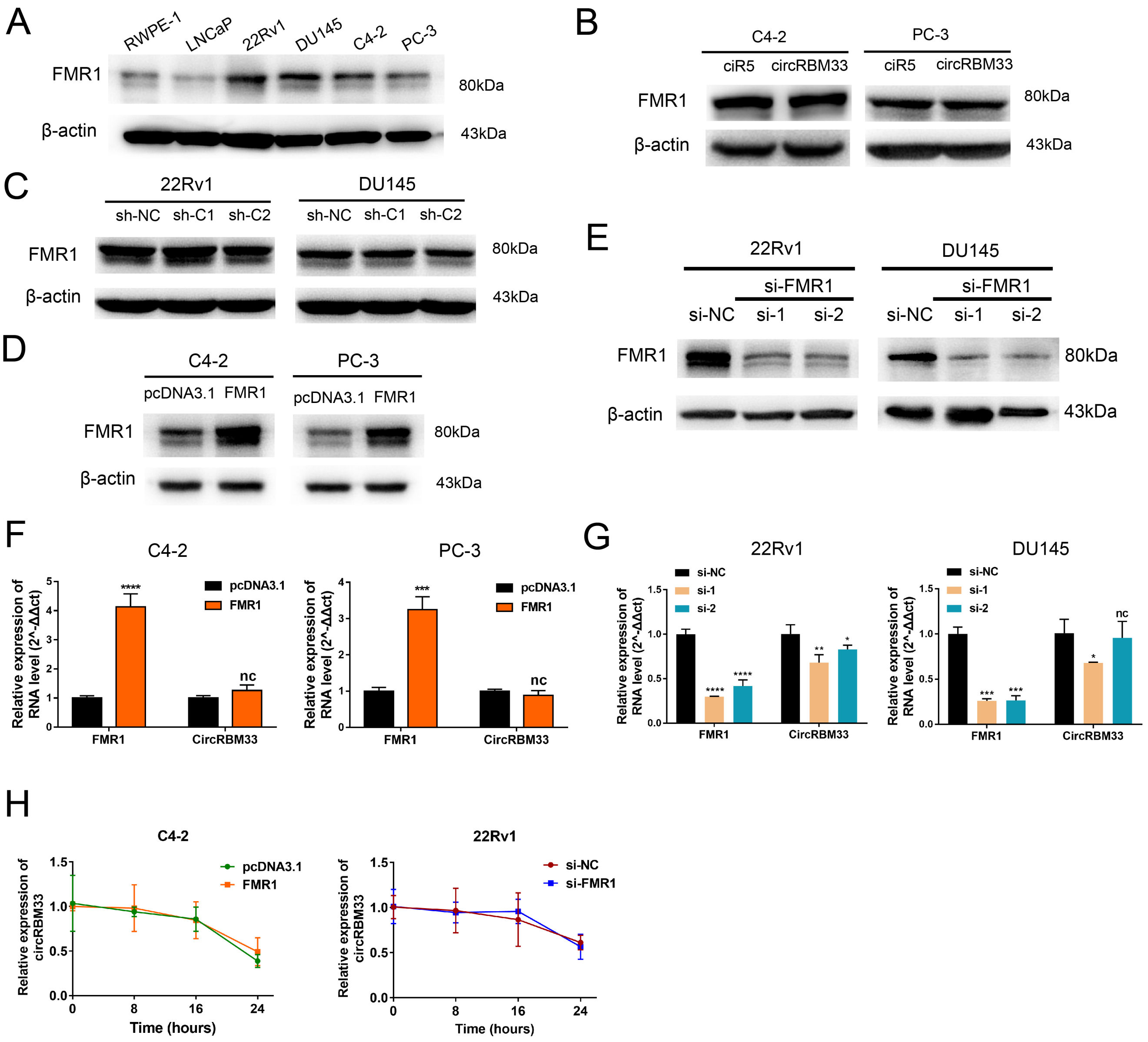

Supplementary Figure 4

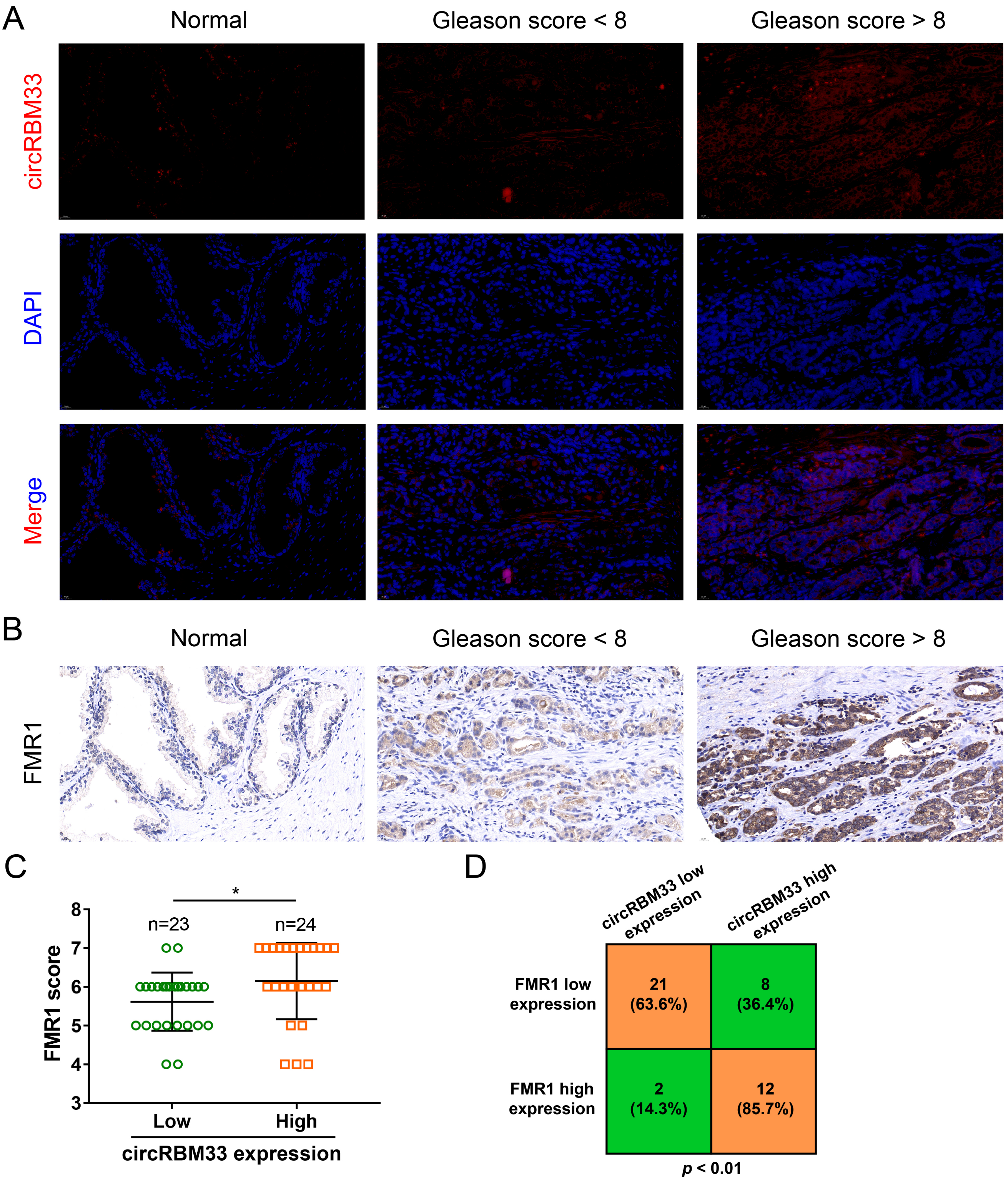

Supplementary Figure 5

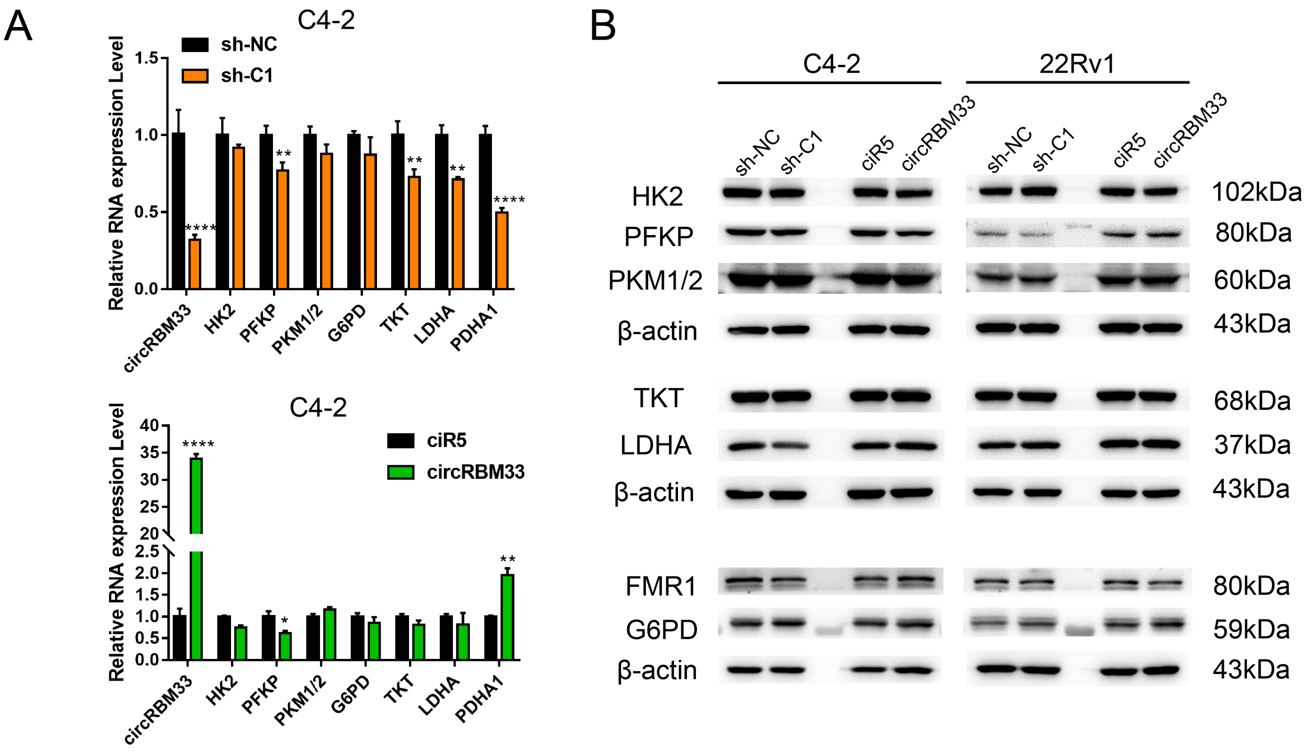

Supplementary Figure 6

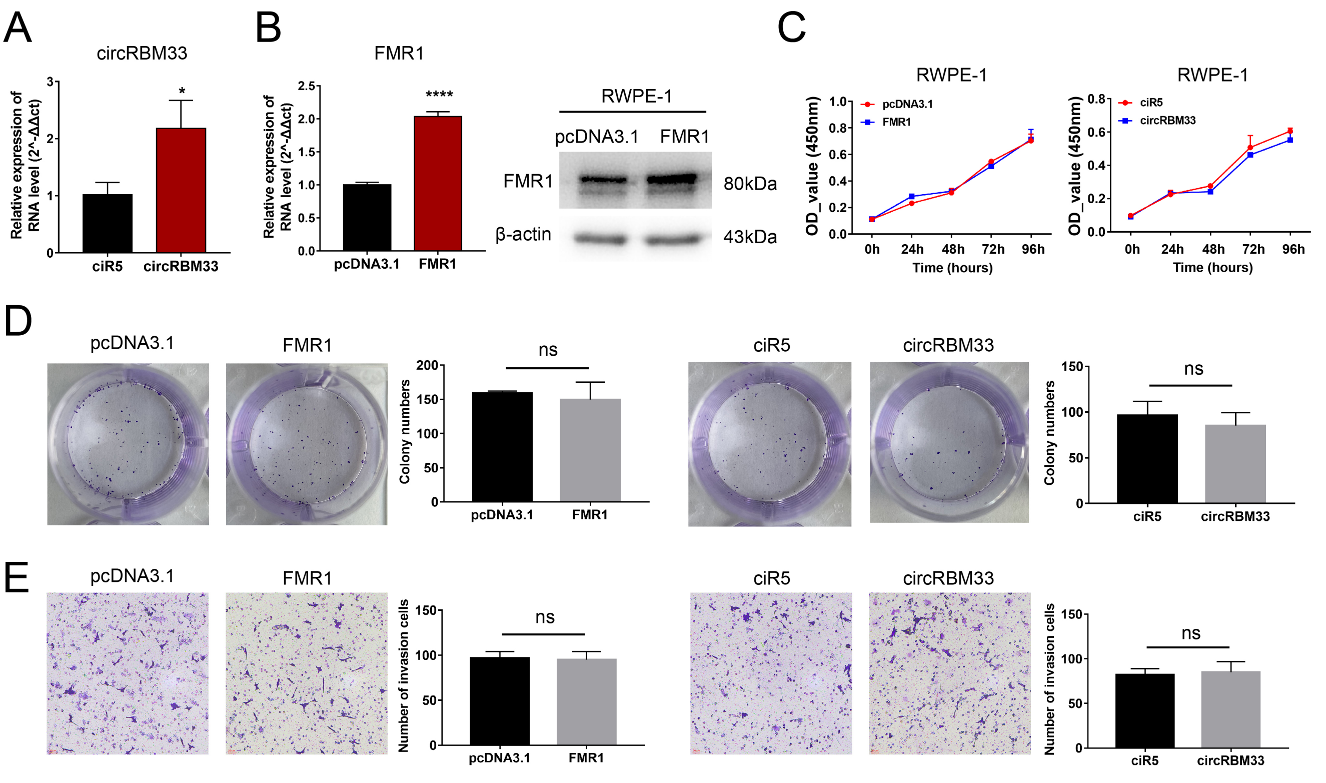

Supplementary Figure 7

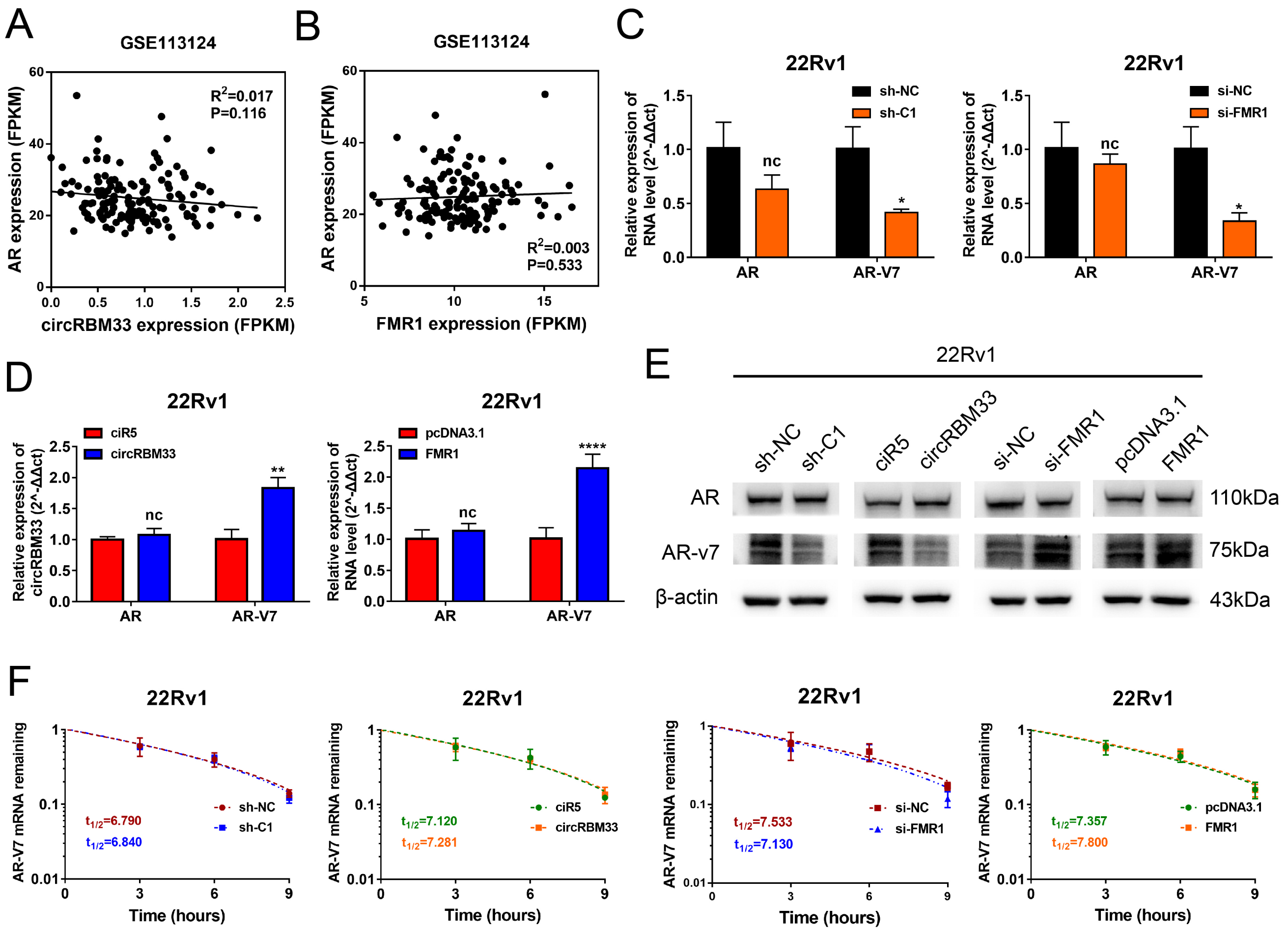

Supplementary Figure 8

Supplementary Table3 Clinicopathological characteristic of circRBM33 in prostate cancer (GSE113124)

	Group	expression	Number	*p* value
Gleason score	>7	1.125±0.417	8	0.231[a]
	=7	0.862±0.431	124	
	<7	0.859±0.251	11	
T_stage	>T2a	1.027±0.528	24	0.054[b]
	≤T2a	0.846±0.391	120	
Preoperative PSA	≥10ng/mL	0.940±0.448	34	0.315[b]
	<10ng/mL	0.857±0.410	110	
Age	≥65	0.923±0.462	42	0.397[b]
	<65	0.857±0.403	102	
Metastasis	Yes	0.871±0.430	9	0.977[b]
	No	0.875±0.423	134	

The expression of circRBM33 was determined by FPKM value; [a] represented *p* value was tested by One-way ANOVA test; [b] represented *p* value was tested by Student's *t* test.
